# The unexpected costs of expertise: evidence from highly specialized physicians

**DOI:** 10.3389/fpubh.2024.1108254

**Published:** 2024-03-04

**Authors:** Yi Cheng

**Affiliations:** Department of Economics, Columbia University, New York, NY, United States

**Keywords:** physician specialization, skill matching, productivity, congestion, newborn

## Abstract

High U.S. spending on health care is commonly attributed to its intensity of specialized, high-tech medical care. A growing body of research focuses on physicians whose medical decisions shape treatment intensity, costs, and patient outcomes. Often overlooked in this research is the assignment of physician skills to patient conditions, which may strongly affect health outcomes and productivity. This matching may be especially important in the case of hospital admissions as high-frequency fluctuations in patient flow make it challenging to maintain effective matches between the best-suited physicians and their patients. This paper focuses on hospitals' responses to demand shocks induced by unscheduled high-risk admissions. I show that these demand shocks result in physician–patient mismatches when hospitals are congested. Specifically, highly specialized physicians who are brought in to treat unscheduled high-risk admissions also treat previously admitted lower-risk patients. This leads to increased treatment intensity for lower-risk patients, which I attribute to persistence in physician practice style. Despite the greater treatment intensity, I find no detectable improvement in health outcomes, which prima facie could be viewed as waste. However, this paper demonstrates that such mismatches mostly happen when the cost of maintaining preferred physician–patient matching is high, which reflects hospitals' conscientious assessment of costs and benefits and should not be simply interpreted as inefficiency. These findings provide vital information for policy-makers looking to identify waste in utilization and create incentives to enhance efficiency in the health care sector.

## 1 Introduction

At 19.7% of its GDP in 2020, the United States spends twice the OECD average on health care. Given its unsatisfactory average health outcomes, there is substantial interest in the effectiveness of U.S. health care spending and whether providers can reduce spending without compromising care quality and health outcomes. Existing research has yet to reach a consensus on the magnitude and sources of waste (1–6). In part, this is due to empirical challenges in measuring productivity and identifying unproductive spending in the health care system.

Although payments to physicians only constitute a small fraction of the aggregate health care **s**pending, physicians' medical decisions clearly shape care utilization and patient outcomes (7). A growing body of research shows that increasing specialization leads to large variation in skills and practice styles among medical professionals, and that specialized physicians tend to adopt more intensive practice styles. However, empirical findings on whether physician specialists, or physicians with more intensive styles, provide higher quality care are mixed (8–13). Existing studies usually evaluate productivity focusing on a constant, physician-specific measure. It is often overlooked that productivity may vary within physicians, depending on the type of patients they are treating. Therefore, the matching between physicians' skills and patients' conditions (“skill–task matching”) can affect both care utilization and patient outcomes, hence constituting a key component in measuring productivity.

This paper examines skill–task matching in hospital admissions. Assessing the impact of physician–patient assignments is challenging. On the one hand, high levels of specialization and large variations in practice styles across medical professionals would increase the gains from matching the best-suited physicians to patients. On the other hand, due to the variability and unpredictability in patient flow, maintaining good physician–patient matchings for every admission can be costly, and even outweigh the benefits of physicians' specialized skills. Previous studies have recognized that fluctuations in patient flow affect care provision and incur costs to both hospitals and patients. Policies have attempted to relieve congestion in order to improve care quality (14–21). However, relatively unexplored is whether the need of matching physician type to patients is an important source of costs. To my knowledge, this paper is the first to provide empirical evidence on skill–task matching stemming from demand uncertainty in the health care sector.

In particular, I analyze how short-term demand fluctuations induced by unscheduled high-risk admissions affect health care production in hospitals. I pay special attention to whether hospitals develop differential responses depending on the level of costs or difficulties in achieving good physician–patient matchings, and how in turn these responses affect health care production. The level of hospital congestion serves as a proxy for the costs of matching in this study. Finding a physician who specializes in treating a certain condition is relatively easy when many physicians are available. But when hospitals become congested, achieving good matchings for every patient may become difficult: more physicians are occupied, and the skill range of available physicians becomes more limited.

Using New York hospital discharge micro data, I focus on newborns. Childbirth is the most common reason for hospitalization in the United States (22) and at-risk newborns disproportionately drive the high aggregate spending on neonatal care (23). Hospital discharge records provide rich information on physicians and patients, treatment decisions, and health outcomes. Additionally, patients' arrivals and assignments to physicians are explicitly recorded in the high-frequency micro data, allowing for the study of skill–task matching and its effects on health care production. Furthermore, effects on newborn health can lead to long term impacts later in life, such as educational attainment, adult disability, and labor market outcomes (24–27).

Birth weight is the most commonly used metric of newborn health both in the literature and in medical practice. Newborns weighing < 1,500 g (“very low birth weight”) require immediate and intensive neonatal care. The precise timing of vaginal deliveries is hard for hospitals to predict. Hence, vaginally-delivered very low birth weight births may serve as demand shocks to hospitals. In this study, I refer to vaginally-delivered very low birth weight infants as “high-risk” unscheduled admissions. Using an event study framework, I find that hospitals summon physicians with more intensive practice styles who specialize in treating high-risk newborns upon unscheduled high-risk admissions. Critical for my purpose, these highly specialized physicians who are called in also treat previously admitted newborns (“incumbent newborns”). This spillover effect is especially pronounced when hospitals are congested, creating exogenous variation in the typical physician–patient matching.

I demonstrate that newborns admitted *prior to* unscheduled high-risk admissions and newborns not affected by any demand shocks do not differ in observables at admission, which supports the exogeneity of my demand shocks. When hospitals are congested, lower-risk newborns admitted just before unscheduled high**-**risk admissions are more likely to be treated by highly specialized physicians, leading to increased treatment intensity. Despite being treated more intensively, little improvement in patient outcomes is seen. This suggests low, even zero, marginal returns to care utilization, indicating that the return to care among mid-risk newborns has reached the “flat-of-the-curve.” Many studies have established that specialists and their intensive practice styles can benefit high-risk patients, which point to a positive return to additional care (10, 11, 28). Results in this study, however, suggest that physician productivity is patient-dependent: physicians who specialize in treating high-risk patients may provide low-return care when treating lower**-**risk patients. Notably, this low return is found for newborns weighing between 1,500 and 2,500 g who are “mid-risk,” i.e., excluding normal birth weight infants. These findings unveil an important source of the “flat of the curve” health care expenditure in the US, i.e., the costs associated with the mismatch between physician experts and patients that best fit their style, and highlight the importance of matching physicians' skills to patients' conditions in health care production.

*Prima facie*, the low productivity resulting from physician–patient mismatch may appear purely wasteful. However, it is worth emphasizing that such low productivity is *only* detectable when hospitals are congested and matching the best-suited physicians to patients is costly. This finding is consistent with predictions from a stylized model: optimal decisions depend on the relative magnitudes of costs and benefits associated with achieving good matchings; allowing a degree of mismatch can be optimal if the matching costs are high. Hence, the mismatch observed at high congestion levels may reflect hospitals' careful assessment of costs and benefits when assigning physicians to patients. Analyses of incumbent newborn characteristics also suggest that hospitals attempt to maintain good physician–patient matchings given the availability of physicians: among mid-risk incumbent infants, newborns with worse health conditions (although still healthier than the high-risk newborns) tend to be assigned to the highly specialized physicians. In more extreme cases, I find that the highly specialized physicians do not treat any incumbent newborns when the expected returns to their specialized skills are too low.

This paper contributes to the literature on physician productivity and health care production. It has been established in the literature that physician specialists spend more (28). However, evidence on its impact on patient health has been mixed (8–11, 28). The evidence on patient-dependent physician productivity presented in this paper provides a possible explanation for the lack of research consensus on how physicians' skills and treatment intensity affect patient outcomes: the productivity response is shaped by which subpopulation of patients are treated and the productivity of highly specialized physicians are not universal but instead task-dependent. This highlights the importance of considering skill–task matching in assessing physician productivity. In addition, by restricting empirical comparisons to be within hospitals, this study isolates the effect of physician practice on care utilization, which complements existing literature on regional or cross-hospital variation in medical spending with empirical evidence that physicians' practice styles contribute to variations in spending among many other factors, such as differences in patient composition or facility quality (6, 9, 12, 29–32). More importantly, findings in this paper demonstrates that the cost associated with the mismatch between physician experts who have more intensive treatment style and patients who are most suitable for the intensive treatment style can be an important source of the “flat of the curve” health care expenditure in the US (30, 33–38).

## 2 Materials and methods

### 2.1 Research setup

This study analyzes the matching of skills to tasks in a highly specialized industry, the health care sector. In the case of hospital admission, patients are typically assigned to physicians based on perceived patient condition and physician expertise. Hence such selection bias usually impedes the empirical evaluation of matching and physician productivity. This paper overcomes this selection bias by exploiting an exogenous variation in physician–patient matching resulting from short-term demand fluctuations. When an unscheduled high-risk patient is admitted, hospitals frequently need to adjust physician resource allocation among existing patients to accommodate the unexpected increases in care demand.

In this study, I focus on the neonatal care sector and investigate hospitals' responses to unscheduled high-risk admissions. I first document the strategies hospitals adopt in adjusting physician–patient matching when an unexpected high-risk newborn is admitted under an event study framework. I then compare newborns admitted just prior to unscheduled high-risk admissions (treated group) to those having little overlap with any unscheduled high-risk admissions (control group) to evaluate the spillover effects of such demand shocks on incumbent newborns. If the unexpected demand shocks are quasi-random in time, newborns in the treated and control groups are expected to be comparable in all aspects upon birth admission. In this case, any differences in subsequent outcomes can be attributed to differences in changes of care provision induced by the unexpected high-risk newborn admissions. Although some may worry that patients may choose instead of randomly assigned to their physicians in the case of childbirth, such selection is unlikely in the case of at-risk newborns, especially among those admitted to NICU upon their births.

I define unscheduled high-risk admissions to be vaginally-delivered very low birth weight newborns, noting first that birth weight has been shown as a good metric of newborn acuity and expected care utilization. Low birth weight newborns, i.e., birth weight below 2,500 g, and especially very low birth weight newborns, i.e., birth weight below 1,500 g, receive special care treatments and utilize a high amount of care resources, hence leading to large increases in demand at hospitals (39).

**Figure 1:**
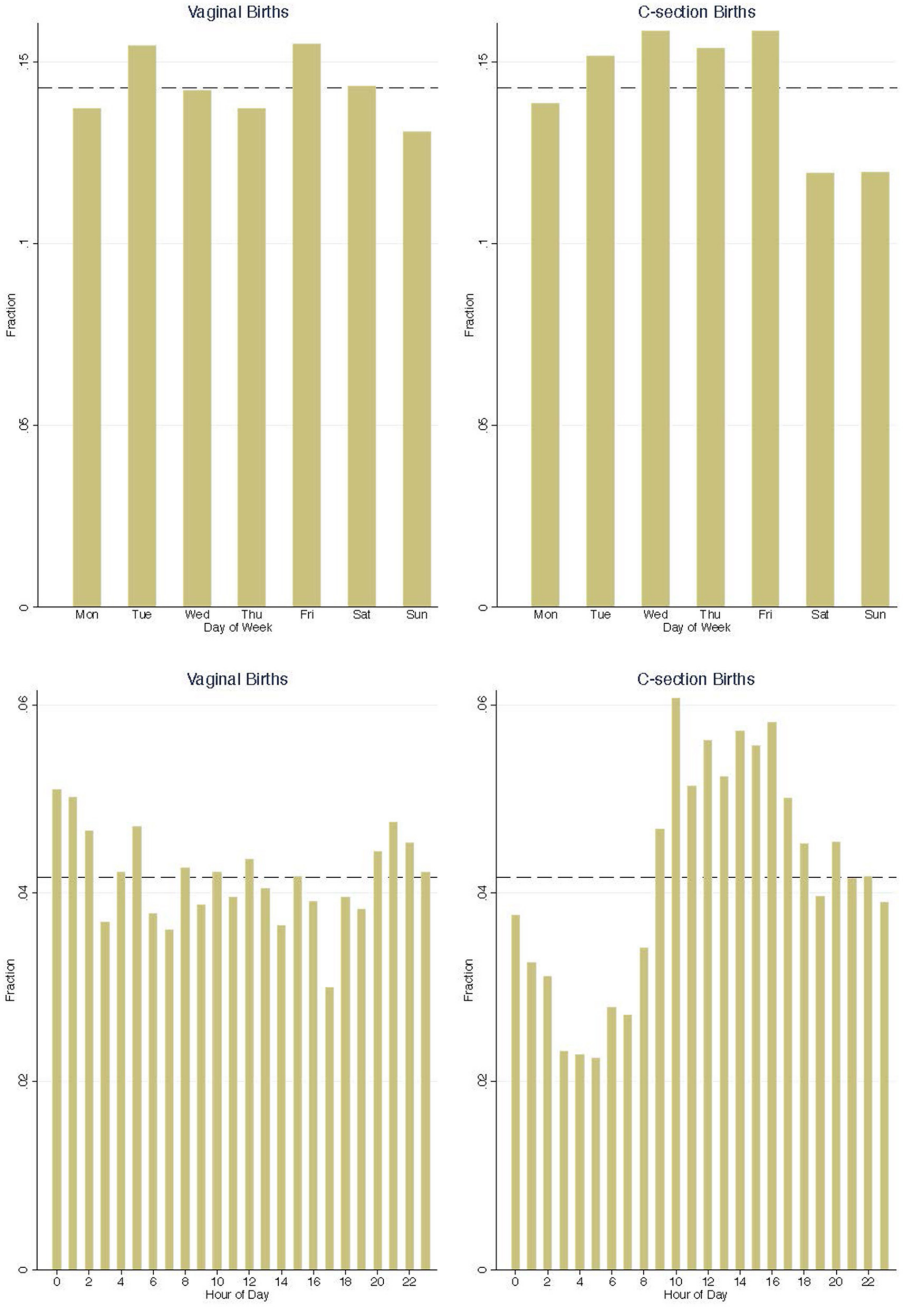
Distribution of high-risk admissions across day of week and hour of day. The dash line is at 1/7 and 1/24, showing an hypothetical uniform distribution.

The quasi-randomness of demand shocks in this study arises from the rareness of high-risk admissions and the lack of predictability of vaginal delivery birth time.^1^ Very low birth weight newborns are only 1.5% of total births and the median time gap between two vaginally-delivered very low birth weight births in my sample is 14 days. Figure 1 show time distributions of high-risk newborn admissions by delivery methods. The left panels suggest that vaginal delivered high-risk admissions are evenly distributed in time. The right panels show decreases in c-section high-risk admissions on weekends and in early mornings. This non-smooth time pattern indicates that at least some c-section high-risk births are scheduled.

Utilizing the quasi-random admission time of vaginally-delivered very low birth weight newborns, I implement an event study to analyze hospitals' responses to unscheduled high-risk admissions. I also exploit this natural experimental setting to study the spillover effects on incumbent newborn patients. By assessing the effects on care utilization and patient outcomes among incumbent newborns, I develop a measure of productivity in medical care provision when facing demand fluctuations.

### 2.2 Hospital discharge data

This study utilizes New York hospital discharge records collected under the Statewide Planning and Research Cooperative System (SPARCS) from 2005 to 2009. SPARCS collect detailed information on patient characteristics, admission and discharge time, diagnoses and treatments, services, and charges for each hospital inpatient stay. I use patient age and principal diagnosis code to identify birth admissions (“newborn sample” thereafter) and their delivery method, and use birth weight to assign newborn acuity level: low birth weight (LBW) as mid-risk admissions and very low birth weight (VLBW) as high-risk admissions. Events of unscheduled high-risk admissions and treated/control groups are defined using patient admission and discharge date and time.

Care utilization is measured by length of hospital stay, total charges, and number of procedures received. Hospital readmission and mortality are used to calibrate patient outcome. The unique patient identifier (UPI) traces medical records of the same patient across hospitals over time. This allows me to measure care utilization and patient outcome beyond one's initial hospital discharge.

Each patient admission is assigned with an attending physician on the discharge record. Physician license information from the New York State Education Department (NYSED) Office of Professions is matched to patient admissions by a unique state physician license identifier. The key physician characteristics used in this study includes (i) physician tenure, calculated based on the date of licensure; (ii) physician specialty or “experience with at-risk newborns,” measured as the fraction of newborn patients being VLBW and LBW in the past; and (iii) physician practice intensity, proxied using raw and residual average total charges, length of stay, and number of procedures among past newborn patients. Detailed sample and variable definitions are described in Sections 2.4 and 2.6 and Supplementary Section 1.

### 2.3 NICU daily census and congestion

I construct a NICU daily patient census to measure the level of congestion. The UB-04 revenue codes in hospital discharge records provide information on the type of accommodation and the number of days of each accommodation one received during the hospital stay. I follow Freedman (19) and flag revenue codes of Nursery Level III (“Intermediate Care”) and Nursery Level IV (“Intensive Care”) as NICU accommodations. The codes are listed in chronological order, which allows me to derive the NICU admission and discharge dates. Using each patient's NICU admission and discharge dates, I derive the number of NICU admissions, NICU discharges, and NICU patient occupancy for each hospital-day.^2^

Daily NICU congestion level is defined based on the quartiles of daily NICU occupancy within each hospital-year. The top quartile hospital-days are coded as high congestion level, the bottom quartile as low congestion level, and the middle two quartiles as medium congestion level. This occupancy measure better captures the level of relative congestion than using the daily number of empty NICU beds, because hospitals can keep “temporary” NICU beds which usually are not shown in official hospital facility reports. Hence, the actual capacity can frequently go beyond the officially reported bed capacity, which is empirically observed in the hospital discharge data.^3^

### 2.4 Sample description

This study focuses on birth admissions in New York City from 2005 to 2009. Forty-six hospitals in the New York City area recorded live birth admissions during the sample period, and only 36 hospitals had NICU birth admissions. I further exclude two hospitals with annual birth admissions below 100. The resulting sample consists of 489,635 newborn birth admissions in 34 New York City hospitals with NICU facilities.

Birth weight provides a good metric of newborn health and expected care utilization. VLBW newborns, defined as high-risk, only make up 1.5% of total births but demand 33% of total newborn care medical spending and have average in-hospital mortality as high as 16.6%. LBW newborns, defined as mid-risk, constitute 7% of total births, consume 22% of newborn care spending, and have higher in-hospital mortality compared to normal birth weight newborns (Supplementary Figures 1–3).

Table 1 reports summary statistics in the full newborn sample and in subsamples by newborn birth weight categories. Care utilization increases dramatically with lower birth weight. However, newborns with lower birth weight still have worse health conditions upon discharge. It's worth highlighting that high-risk newborns demand intensive care after birth. They on average have a NICU admission rate of 96.2%, stay in hospital for 49.7 days, incur total charges of $263.4k, but still show a 16.6% in-hospital mortality and a 14.1% 1-year readmission rate. Table 1 Panel B reports average attending physician characteristics. Newborns with lower birth weight are treated by physicians with longer tenure, more experience with high-risk newborns, and higher care utilization (Supplementary Figures 4–7).

**Table 1:** Summary statistics.

	**(1)**	**(2)**	**(3)**	**(4)**
**Sample**	**All**	**Low-risk**	**Mid-risk**	**High-risk**
**Panel A: care utilization and patient outcomes**				
Length of stay	3.917	2.791	8.887	49.709
(Median)	2	2	4	45
Total charges	12,272	6,050	40,118	263,435
(Median)	3,813	3,638	11,940	180,016
Number of procedures	1.693	1.528	2.706	7.114
NICU admission	0.158	0.115	0.564	0.962
Death in hospital	0.003	0.000	0.006	0.166
Hospital transfer	0.004	0.002	0.013	0.091
28-day readmission	0.013	0.013	0.016	0.008
1-year readmission	0.043	0.040	0.068	0.141
**Panel B: attending physician characteristics**				
Physician tenure	16.841	16.811	17.118	17.405
Physician experience with VLBW	0.017	0.013	0.050	0.101
Physician experience with LBW	0.072	0.065	0.136	0.204
Physician average length of stay	3.796	3.512	6.314	9.773
(Median)	2.709	2.668	4.134	7.968
Physician average total charges	10,937	9,179	26,455	48,195
(Median)	4,990	4,869	7,549	27,723
Physician average number of procedures	1.582	1.521	2.132	2.808
Observations	489,635	449,029	33,158	7,448

Low-risk sample consists of newborns with birth weight of 2,500 g and above. Mid-risk sample consists of newborns with birth weight between (1,500, 2,500) g, i.e., LBW. High-risk sample consists of newborns with birth weight below 1,500 g, i.e., VLBW.

Median is reported for length of stay and total charges only because the distribution is heavily skewed. Mean and median are close for other continuous variables.

### 2.5 Hospital response: event study specification

I study hospitals' responses under an event study framework at the hospital-day level. Any hospital-day with a vaginally-delivered VLBW newborn admission is flagged as an “event.” There are 2,273 vaginally-delivered VLBW newborn admissions in the sample, resulting in 2,156 events. Overlapping event windows are allowed and multiple event day indicators are assigned to the same hospital-day in such case.


(1) $$\begin{array}{l} Y_{h,t}=\overset{5}{\underset{\text{j}=-5}{\sum }}\phi^{\text{j}}{\text{D}}_{\text{h},\text{t}}^{\text{j}}+\tau_{\text{h},\text{y}}+\tau_{\text{dow}}+\tau_{\text{mm}}+\epsilon_{\text{h},\text{t}} \end{array}$$

- *Y*_*h, t*_ is the outcome of a newborn admission in hospital h on day t.
- $D_{h,t}^{j}$ are event time indicators: $D_{h,t}^{j}=$ 1 for being j days apart from an event. The day before an event is taken as the reference period, i.e., $D_{h,t}^{-1}$ is omitted and ϕ^−1^ is normalized to zero.
- τ_*h, y*_, τ_*mm*_, and τ_*dow*_ are fixed effects for admission hospital-year, month, and day-of-week.

Event study coefficients ϕ^*j*^ capture hospitals' responses to an unscheduled high-risk admission in a 5- day window centered at the day of event. Coefficient on the day of event, ϕ^0^, captures any spontaneous responses to the unscheduled high-risk admission. Post-event coefficients ϕ^*j*^, j > 0, measure any lasting effects or delayed adjustments. Pre-event coefficients ϕ^*j*^, j < 0, provide a test of exogeneity: any pre-event effects would suggest that the admission decisions of vaginally-delivered high-risk newborns may experience some endogeneity.

### 2.6 Spillover effect of unscheduled high-risk admissions: regression specification

To estimate the spillover effects of high-risk admissions on previously admitted newborn patients, I follow the definition in event study and use vaginally-delivered VLBW newborn admissions as unexpected high-risk admissions. C-section VLBW newborn admissions are not used as demand shocks in this section, because they do not distribute smoothly over time as shown in Figure 1. I focus on mid-risk newborns in measuring the spillover effects for two reasons: (1) mid-risk newborns demand more medical care upon birth than healthy newborns and are hence vulnerable to demand shocks; and (2) mid-risk newborns have higher rate of NICU admission, and are hence more likely to share medical resources with high-risk newborns in the NICU. Low-risk healthy newborns mostly stay in the regular nursery, which is physically separate from NICU, after birth and require little medical care. They serve as a placebo group in this study, since unscheduled high-risk admissions are expected to have little impact on these healthy newborns. All multiple births are excluded from the analysis sample for clearer results interpretation.

The analysis sample consists of 23,791 newborn admissions whose birth weight fall between 1,500 and 2,500 g. Newborns admitted within 2 days prior to an unscheduled high-risk admission are assigned to the treated group. The control group consists of all newborns whose birth admission is 3 or more days apart from any unscheduled high-risk admissions.^4^ Adopting an admission time cutoff in assigning newborns to treated and control groups, i.e., a 2-days threshold in this study, is essential. Using actual overlaps with unscheduled high-risk newborns will incur bias or fail to capture key impacts for several reasons: (1) mid-risk newborns only have high demand for care in the first few days, hence are unlikely to be affected if encountering unscheduled high-risk admissions late during their hospital stay, (2) the length of day used to determine overlap is an outcome that could be affected by the unscheduled high-risk admission hence is endogenous, and (3) newborns with longer length of stay tend to have worse health conditions and have higher probability of encountering unscheduled high-risk admissions. The admission time cutoff of 2 days is chosen to best capture the spillover effect: Birth admissions within the 2 days prior to an unscheduled high-risk admission almost surely have some overlap with the high-risk newborn (Supplementary Figure 8), because more than 98% of low birth weight newborns stay in hospital for 2 days and more (Supplementary Figure 9). On the other hand, care intensity is concentrated in the first 3 days during one's hospital stay for mid-risk newborns (Supplementary Figure 10). Hence, adopting a 2-day threshold will capture majority of the spillover effects on care utilization among incumbent newborns.

**Figure 2:**
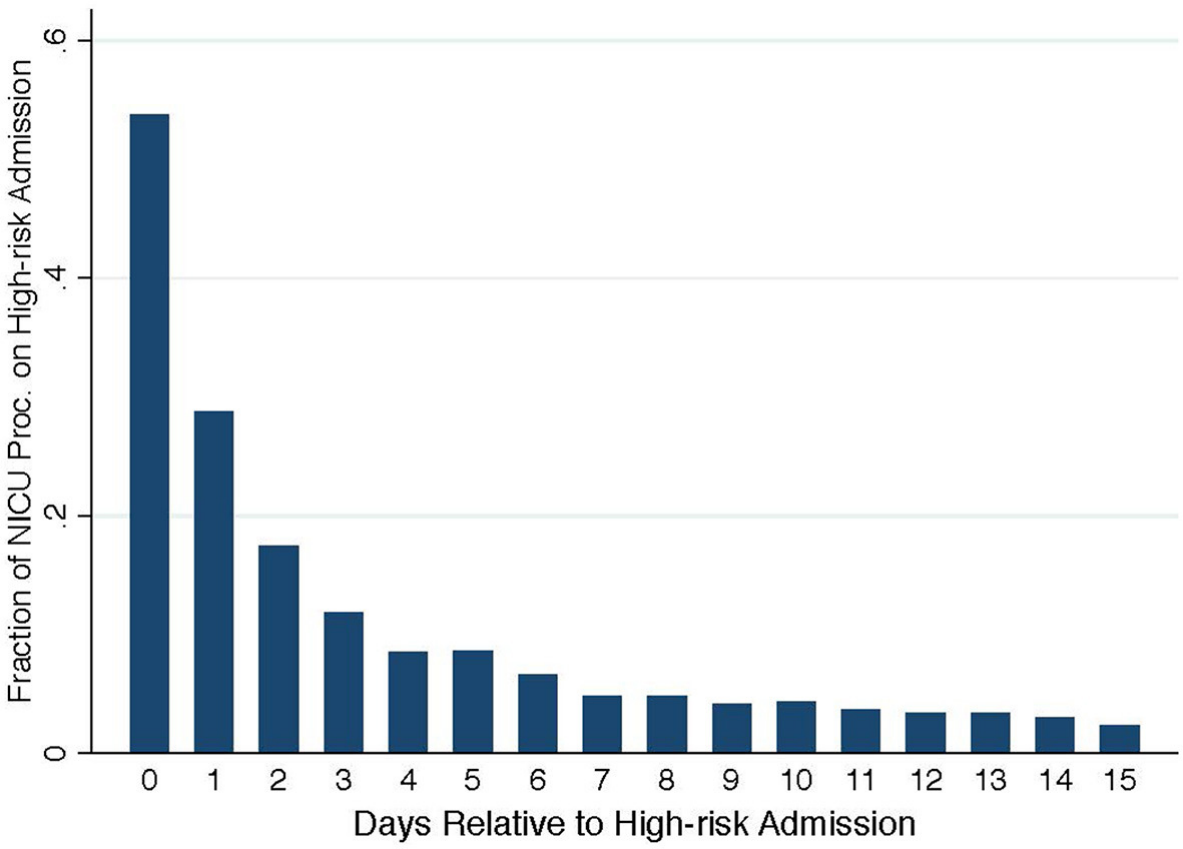
Fraction of procedures on high-risk admissions.

To establish direct evidence that unscheduled high-risk admissions lead to sharp increases in care demand, Figure 2 plots the fraction of NICU treatment procedures that are performed on the unscheduled high-risk newborns. On the day of unscheduled high-risk admission, the newly admitted high-risk newborns take up more than 50% of total NICU procedures. This fraction decreases to ~10% on the 3rd day after birth and further to below 5% on the 7th day and thereafter. Hence, the effects of unscheduled high-risk admissions on subsequent newborn admissions, if there is any, would have largely diminished in the control group who are admitted at least 3 days after.

The key identifying assumption is that encountering an unscheduled high-risk admission within 2 days after birth is random. If this assumption holds true, newborns in the treated and control groups should look similar upon admission. Table 2 presents covariates balance in the sample. Columns 1 and 2 report covariate averages in the control and treated groups. Columns 3–6 report average covariate differences between the treated and control groups, controlling for hospital-year, month, and day-of-week fixed effects. All differences are small and insignificant in the full sample and at each congestion level. This provides strong support that the treated–control status is as good as randomly assigned. Therefore, any difference in care utilization and patient outcomes among incumbent newborns serves as a measure of spillover effect.

**Table 2:** Covariates balance table.

	**(1)**	**(2)**	**(3)**	**(4)**	**(5)**	**(6)**
	**Control**	**Treated**	**Treated-control difference**	**Treated-control difference**	**Treated-control difference**	**Treated-control difference**
C-section	0.409	0.392	−0.0152	−0.0137	−0.0137	−0.0270
			(0.0123)	(0.0186)	(0.0198)	(0.0223)
Birth weight	2197.2	2184.0	−5.043	−4.055	−0.625	−16.24
			(6.373)	(12.27)	(10.03)	(13.72)
White	0.258	0.295	−0.00481	−0.00684	−0.00138	−0.0229
			(0.00957)	(0.0148)	(0.0129)	(0.0185)
Black	0.333	0.321	−0.00363	−0.00826	−0.00342	0.00152
			(0.0115)	(0.0233)	(0.0132)	(0.0273)
Female	0.534	0.528	−0.00888	−0.0167	−0.0189	0.0270
			(0.00822)	(0.0187)	(0.0130)	(0.0261)
Medicaid	0.611	0.599	0.00387	−0.00344	0.00360	0.0135
			(0.00760)	(0.0166)	(0.0117)	(0.0191)
Observations	21,629	2,162	23,791	7,015	11,790	4,986
Congestion	All	All	All	Low	Medium	High

Standard errors in parentheses. Standard errors are clustered at hospital level.

Hospital-year, month, and day of week fixed effects are included in measuring treated–control differences.

The patient level regression is specified as follow:


(2) $$\begin{array}{l} Y_{i,h,t}=\alpha \cdot \text{P}{\text{re}}_{\text{i},\text{h},\text{t}}+\beta {\text{X}}_{\text{i},\text{h},\text{t}}+\tau_{\text{h},\text{t}}+\tau_{\text{dow}}+\tau_{\text{mm}}+\epsilon_{\text{i},\text{h},\text{t}} \end{array}$$

- **Y**_*i, h, t*_ is the outcome measure of newborn i admitted on day t in hospital h.
- *Pre*_*i, h, t*_ is the treated group indicator: *Pre*_*i, h, t*_ = 1 for newborns admitted within 2 days prior to an unscheduled high-risk admission.
- *X*_*i, h, t*_ flexibly controls for patient observables, including indicators for birth delivery method, insurance type, race, gender, and birth weight (250-g bins).
- τ_*h, y*_, τ_*mm*_, and τ_*dow*_ are hospital-year, birth month, and day-of-week fixed effects.

α is the coefficient of interest in this study, which captures any differences between the treated and control groups. It measures the spillover effect if the outcome variable is care utilization or patient outcome. When substituting patient observables as outcome variables in the regression, coefficient provides a direct test of the identifying assumption.

To examine the effect heterogeneity across congestion levels, I interact the treated group indicator with the congestion indicator as follow:


(3) $$\begin{array}{l} Y_{i,h,t}=\sum_{\text{c}}\alpha_{\text{c}}\cdot {\text{Pre}}_{\text{i},\text{h},\text{t}}\cdot 𝟙\left({\text{C}}_{\text{h},\text{t}}\text{=c}\right)\text{+}\beta {\text{X}}_{\text{i},\text{h},\text{t}}\text{+}\sum_{\text{c}}\gamma_{\text{c}}\text{+}\tau_{\text{h},\text{t}}\\ \;+\tau_{dow}+\tau_{mm}+\epsilon_{i,h,t} \end{array}$$

- *C*_*h, t*_ is the NICU congestion indicator described in Section 2.3.
- α_*c*_ measures spillover effects at each congestion level.
- γ_*c*_ captures base level effect of hospital congestion.

To allow flexibility, I implement an augmented regression model by interacting the congestion indicator with all covariates and fixed effects in Equation 3. The augmented regression is equivalent to subsample regressions at each congestion level following Equation 2. Coefficient estimates with and without covariates-congestion interactions are both reported for comparison.

## 3 Results

### 3.1 Hospital response to unscheduled high-risk admissions

Unscheduled high-risk admissions, i.e., vaginally-delivered VLBW births, lead to demand shocks in hospitals. In this section, I investigate how hospitals respond to unscheduled high-risk newborn admissions and explore whether the response varies by the level of congestion, measured by NICU patient occupancy.

**Figure 3:**
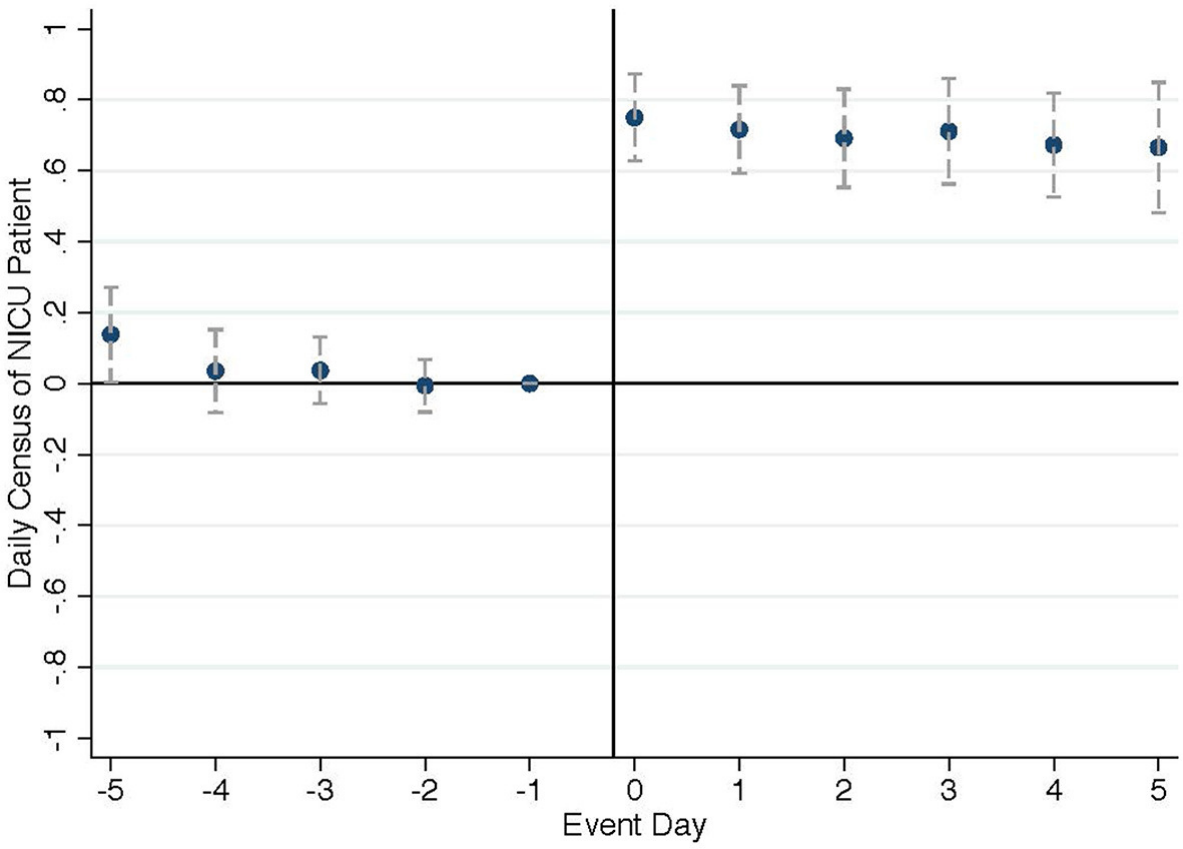
Effect on NICU occupancy.

Figure 3 plots event study coefficients on daily NICU occupancy, showing a spontaneous and lasting increase. The number of NICU patients increases by 0.75 on the day of event and persists for at least 5 subsequent days. Analyzing the NICU admission and discharge patterns, the increase in NICU occupancy is driven by the NICU admission of the unscheduled high-risk newborn, which is consistent with the high NICU admission rate for VLBW newborns. The NICU discharge also increases slightly on the event day driven by same-day discharges of the unscheduled high-risk newborn themselves (Supplementary Figure 11).^5^ In addition, we might expect hospitals to discharge patients early or reduce subsequent admissions to manage the demand shock, especially when NICU occupancy is high. However, NICU discharge patterns show no sign of such behavior (Supplementary Figure 12). This suggests that hospitals possess some degrees of flexibility in physical capacity to accommodate short-term demand fluctuations even when the occupancy level is high, consistent with the empirical observation that NICU occupancy can frequently exceed their registered bed capacities.

**Figure 4:**
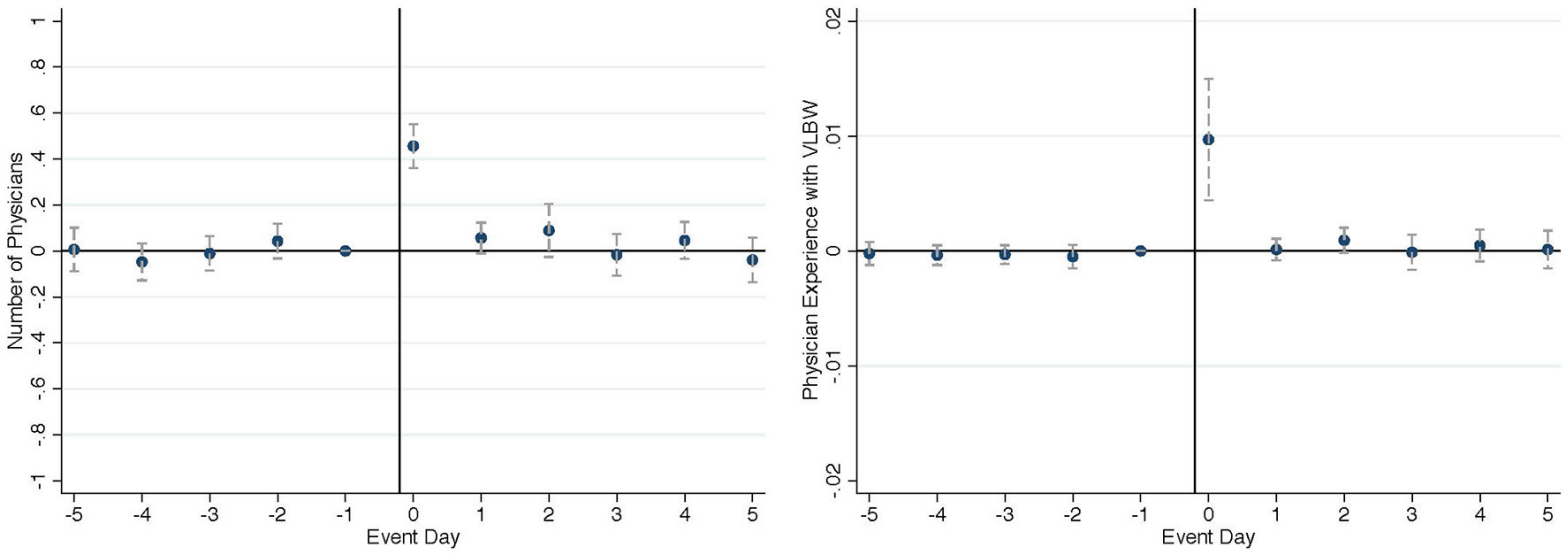
Effect on physician assignment (**left**: number of physicians on duty, **right**: physician specialization).

Turning to physicians, I investigate how hospitals manage physicians in response to unscheduled high-risk admissions. Figure 4 left panel points to an increase in the number of attending physicians on duty when unscheduled high-risk newborns are admitted. The right panel indicates that hospitals not only increase the physician count, but also increase the level of physician specialization in the event of an unscheduled high-risk admission.

**Figure 5:**
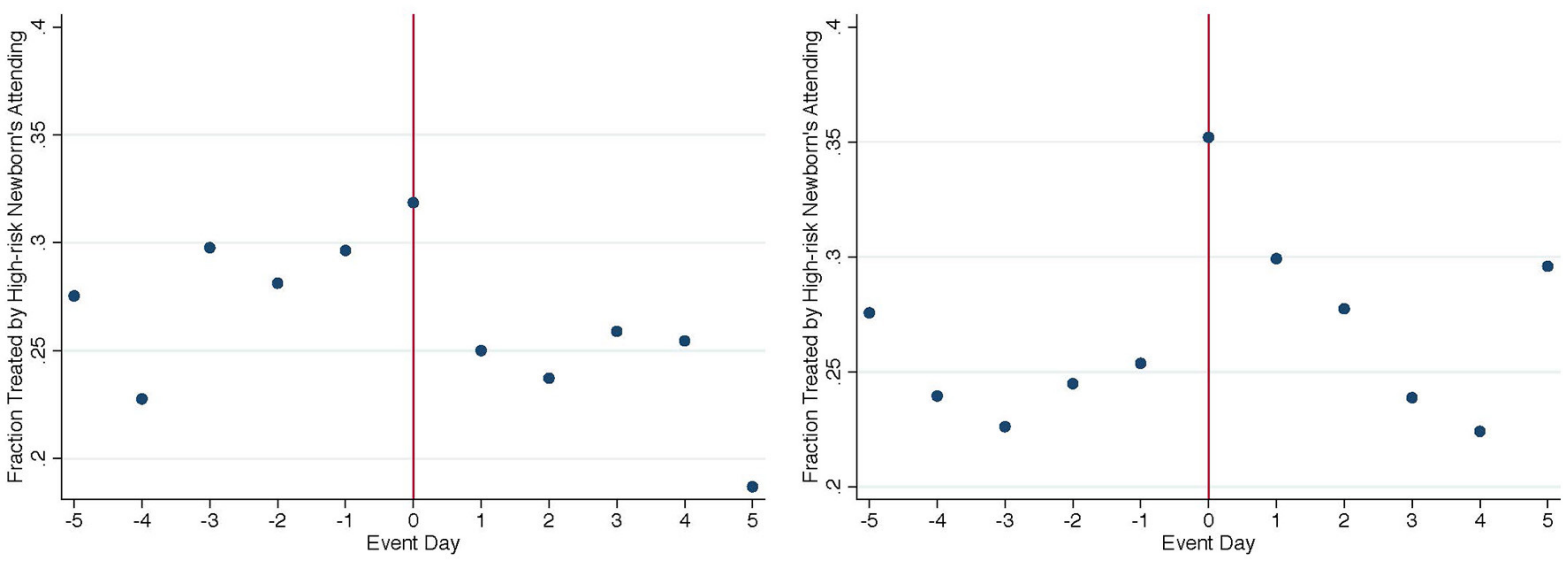
Fraction of mid-risk newborns treated by specialized physicians (**left**: high congestion, **right**: low congestion).

The event study results suggest that unscheduled high-risk newborn admissions draw in additional resources, such as highly specialized physicians, instead of purely competing for existing resources with previously admitted newborns. The increase in available resources likely will not only be allocated to high-risk newborns alone, but also affect incumbent newborns as well. Figure 5 plots the fraction of mid-risk newborn admissions attended by the attending physicians of (“specialized physicians”) on each day in the 5-day window conditional on NICU congestion level. When NICUs are congested, the highly specialized physicians attending high-risk newborns help treat ~30% incumbent mid-risk newborns admitted 2–3 days prior, while the likelihood of treating incumbent mid-risk newborns are much lower when NICUs are not congested.^6^ The differential response in physician assignment across congestion levels points to further spillover effects on care utilization and patient outcomes among incumbent newborns. In the next section, I quantify these spillover effects by NICU congestion levels and draw insights into medical care productivity based on the empirical findings.

### 3.2 Spillover effect of unscheduled high-risk admissions

Event study results in Section 3.1 indicate that unscheduled high-risk admissions can affect physician assignment among previously admitted newborns, especially when the NICU congestion level is high. This exogenous variation in physician–patient matching provides a natural experimental setting to study the effect of physician practice on patient outcomes. In this section, I implement patient level regression analyses among incumbent newborns and report estimated spillover effects on physician practice styles, care utilization, and patient outcomes. Results in this section indicate that incumbent newborns receive more intensive treatments but show little health improvement. The increase in care utilization is likely driven by physician practice styles.

In theory, when the congestion level is high, hospitals face a higher need to call in specialized physicians and assign incumbent patients to the specialized physicians. This in turn would result in a spillover in the use of medical resource among incumbent patients. When hospitals are less congested, a crowding out effect is more likely to occur among incumbent newborns in the event of unscheduled high-risk admissions, because hospitals would be reluctant to bring in additional physicians and existing medical resources utilization would concentrate more on the high-risk patients.

In this section, I report the estimates of spillover effects from regression Equations 2, 3 and show that the above hypothesis is well-supported by the empirical evidence. Two sets of outcomes are analyzed: (1) attending physician profile and practice style; and (2) care utilization and patient outcomes. In Tables 3–**6**, regression coefficients from Equation 2 in the full sample are reported in the top panel and estimated effects at each congestion level are reported in the bottom panel. The effect estimates attain similar magnitudes with or without congestion-covariates interactions. Hence, I only report estimates with congestion-covariates interactions from subsample regression when discussing alternative specifications and robustness checks.

**Table 3:** Differences in attending physician profile.

	**(1)**	**(2)**	**(3)**	**(4)**	**(5)**	**(6)**
**Congestion**	**Physician tenure**		**Experience with VLBW**		**Experience with LBW**	
All	−0.234		0.00138		−0.0000981	
	(0.258)		(0.00146)		(0.00186)	
Low	−0.882^**^	−0.824^*^	−0.000221	−0.000908	−0.00448	−0.00619^*^
	(0.380)	(0.408)	(0.00323)	(0.00317)	(0.00338)	(0.00340)
Medium	−0.114	−0.0789	0.000517	0.000225	−0.00161	−0.00177
	(0.316)	(0.352)	(0.00279)	(0.00295)	(0.00221)	(0.00221)
High	0.406	0.423	0.00594^*^	0.00606^*^	0.0101^**^	0.0117^***^
	(0.427)	(0.514)	(0.00335)	(0.00314)	(0.00418)	(0.00405)
Covariate x C	No	Yes	No	Yes	No	Yes
*N*	23,164	23,164	23,669	23,669	23,669	23,669
Y-mean	17.13	17.13	0.0459	0.0459	0.130	0.130

Standard errors in parentheses. Standard errors clustered at hospital level. ^*^p < 0.1, ^**^p < 0.05, ^***^p < 0.01.

VLBW newborns: high-risk newborns; LBW newborns: mid-risk newborns.

A small fraction of patients have no attending physician measures because of missing physician license information or no previous admitted patients to construct experience measure.

#### 3.2.1 Attending physician profile and practice styles

As discussed at the beginning of this section, newborns admitted prior to unexpected high-risk admissions may experience either positive or negative spillovers in terms of physician–patient matching. This section summarizes the spillover effect in terms of attending physician tenure, specialization, and practice style.

Table 3 reports differences in attending physician profile. Coefficients in the top panel indicate no overall difference between the treated and control groups. The bottom panel suggests that when NICUs are congested, newborns admitted within the 2 days prior to unscheduled high-risk admissions are attended by physicians more experienced in treating sick newborns. Columns 3–6 indicate that treated group newborns are assigned to physicians with 6% more experience with high-risk newborns and 10% more experience with mid-risk newborns at a high congestion level. When the NICU occupancy is low, columns 1 and 2 indicate that newborns admitted before unscheduled high-risk admissions are treated by more junior physicians, shown by a decrease in tenure of 0.9 years. These physicians are also marginally less specialized in treating sick newborns.

Table 4 reports differences in attending physician practice style. The top panel indicates an overall more intensive style shown by higher average number of procedures. Coefficients in the bottom panel indicate that this effect is driven by the high congestion level. Incumbent newborns are treated by physicians who, on average, assigning 4% longer length of stay, 7% higher charges, and 4% (0.0849/2.085) more treatment procedures when NICU facilities are congested.^7^

**Table 4:** Differences in attending physician practice style.

	**(1)**	**(2)**	**(3)**	**(4)**	**(5)**	**(6)**
**Congestion**	**Avg. length of stay (log)**		**Avg. total charges (log)**		**Avg. # of procedures**	
All	0.000803		0.00638		0.0326^**^	
	(0.00736)		(0.0144)		(0.0157)	
Low	−0.0172	−0.0277^*^	−0.0304	−0.0502	0.0206	0.00142
	(0.0170)	(0.0161)	(0.0324)	(0.0304)	(0.0283)	(0.0282)
Medium	−0.00335	−0.00342	0.00628	0.00531	0.0150	0.0140
	(0.00934)	(0.00952)	(0.0202)	(0.0209)	(0.0202)	(0.0210)
High	0.0364^*^	0.0409^**^	0.0597	0.0708^**^	0.0889^**^	0.0849^**^
	(0.0203)	(0.0188)	(0.0377)	(0.0346)	(0.0373)	(0.0348)
Covariate x C	No	Yes	No	Yes	No	Yes
*N*	23,535	23,535	23,535	23,535	23,535	23,535
Y-mean	1.771	1.771	9.323	9.323	2.085	2.085

Standard errors in parentheses. Standard errors clustered at hospital level. ^*^p < 0.1, ^**^p < 0.05, ^***^p < 0.01.

For patients admitted on day t attended by physician p, physician practice style is measured as average total charges, length of stay, and number of procedures among newborn patients discharged by physician p up to day t-1.

A small fraction of patients have missing physician practice measures because there is no previously discharged patients by their attending physicians.

Tables 3, 4 indicate that the differential effects on attending physician profile at different congestion levels are consistent with our expectation. When NICU congestion level is low, incumbent newborns experience a crowding out effect and are treated by physicians with shorter tenure, less experience with at-risk newborns, and less intensive practice styles. When NICU is more congested, the effect estimates indicate the opposite: incumbent newborns are treated by physicians with longer tenure, more experience with at-risk newborns, and more intensive practice styles.

#### 3.2.2 Care utilization and patient outcomes

Section 3.2.1 provides evidence that unscheduled high-risk admissions affect the attending physician assignment among previously admitted newborns. Considering the large influence physicians have on medical decisions, it is likely that care utilization and patient outcome will also be affected. Table 5 summarizes coefficient estimates on length of say, total charges, and number of treatment procedures. The top panel indicates no overall difference between the treated and control group newborns. When focusing on estimates at each congestion level, the bottom panel shows that unscheduled high-risk admissions lead to a 7.31% increase in length of stay, a 10.7% increase in total charges, and an 7.76% (0.208/2.681) increase in number of procedures among incumbent newborns when NICU facilities are congested. To report the effect magnitudes in levels, there is an increase of 0.6 (8.063 × 7.31%) day in length of stay, $3,653 ($34,139 × 10.7%) in total charges, and 0.2 in number of procedures. When NICU congestion level is low, the point estimates indicate a reduction in care utilization, although the effects are smaller and insignificant. To further examine the spillover effects on care utilization, I estimate the effect on cumulative care utilization during the 1st year after birth and obtain estimates similar to Table 5.^8^ This indicates that the increase in care utilization upon birth does not reduce subsequent utilization, and therefore is not a reallocation of care over time within the 1st year of life. The results on care utilization, especially the heterogeneity across congestion levels, are consistent with the findings on physician practice style. This implies that the changes in treatment intensity could be mostly driven by physician practice styles. I provide more discussion on potential mechanisms in Supplementary Section 2.

**Table 5:** Differences in care utilization during hospital stay.

	**(1)**	**(2)**	**(3)**	**(4)**	**(5)**	**(6)**
**Congestion**	**Length of stay (log)**		**Total charges (log)**		**# of procedures**	
All	0.00623		−0.00373		0.0177	
	(0.00984)		(0.0155)		(0.0472)	
Low	−0.0150	−0.0167	−0.0125	−0.0287	−0.0670	−0.0533
	(0.0250)	(0.0241)	(0.0349)	(0.0359)	(0.0573)	(0.0553)
Medium	−0.00638	−0.00510	−0.0399^*^	−0.0362	−0.00764	−0.0145
	(0.0138)	(0.0139)	(0.0231)	(0.0233)	(0.0752)	(0.0705)
High	0.0655^***^	0.0731^***^	0.0913^**^	0.107^**^	0.198^*^	0.208
	(0.0225)	(0.0264)	(0.0435)	(0.0444)	(0.109)	(0.125)
Covariate x C	No	Yes	No	Yes	No	Yes
*N*	23,791	23,791	23,791	23,791	23,791	23,791
Y-mean	1.875	1.875	9.437	9.437	2.681	2.681

Standard errors in parentheses. Standard errors clustered at hospital level. ^*^p < 0.1, ^**^p < 0.05, ^***^p < 0.01.

Whether increased care utilization leads to improvement in patient outcomes? The answer to this question provides a measure of productivity. Table 6 summarizes effect estimates on in-hospital mortality, hospital transfer, and readmissions.^9^ None of the outcome shows any effect, suggesting no clear improvement in patient outcomes. One may criticize that these outcome measures are rare events and it might be underpowered to capture any meaningful effects. However, it is worth noting that this analysis focuses on mid-risk newborns, who have higher likelihood of having adverse health conditions than an average healthy newborn. The point estimates in Table 6 are insignificant and small in magnitudes. If anything, the point estimates on these adverse outcomes at the high congestion level, where we expect to see health improvement, are mostly positive.

**Table 6:** Differences in patient outcomes.

	**(1)**	**(2)**	**(3)**	**(4)**	**(5)**	**(6)**	**(7)**	**(8)**	**(9)**	**(10)**
**Congestion**	**In-hospital mortality**		**1-year hospital mortality**		**Hospital transfer**		**28-day readmission**		**1-year readmission**	
All	0.00159		0.00110		0.000381		−0.00175		0.00703	
	(0.00239)		(0.00272)		(0.00157)		(0.00330)		(0.00647)	
Low	0.00734	0.00593	0.00633	0.00553	−0.00395	−0.00346	−0.00516	−0.00639	−0.00140	0.000309
	(0.00727)	(0.00650)	(0.00715)	(0.00648)	(0.00356)	(0.00413)	(0.00392)	(0.00495)	(0.00788)	(0.00810)
Medium	−0.00247	−0.00200	−0.00228	−0.00176	0.00125	0.00171	−0.000813	−0.000460	0.00991	0.0104
	(0.00184)	(0.00192)	(0.00191)	(0.00212)	(0.00278)	(0.00269)	(0.00565)	(0.00516)	(0.00880)	(0.00792)
High	0.00291	0.00529	0.00155	0.00314	0.00458	0.00183	0.00115	−0.000393	0.0126	0.00476
	(0.00441)	(0.00485)	(0.00434)	(0.00488)	(0.00708)	(0.00759)	(0.00640)	(0.00725)	(0.0189)	(0.0195)
Covariate x C	No	Yes	No	Yes	No	Yes	No	Yes	No	Yes
*N*	23,791	23,791	23,791	23,791	23,791	23,791	23,791	23,791	23,791	23,791
Y-mean	0.00706	0.00706	0.00782	0.00782	0.0130	0.0130	0.0171	0.0171	0.0699	0.0699

Standard errors in parentheses. Standard errors clustered at hospital level.

The findings of increased treatment intensity and lack of observable health benefits imply a low or zero return to the additional care utilization among incumbent newborns. It is likely that the level of care provision among mid-risk newborns has reached the “flat-of-the-curve.” Therefore, the more intensive practice styles of highly specialized physicians do not generate noticeable patient benefits. One may interpret this finding as an indication of low physician productivity, and that assigning highly specialized physicians to lower-risk newborns when hospitals are congested purely results in wasteful medical spending. However, such a conclusion ignores the costs to achieve a seemingly more efficient physician–patient matching. When hospitals are congested, additional physicians will be needed to reduce waiting and ensure care quality. In the case of no high-risk newborn admissions, hospitals may bring in additional physicians normally treating mid-risk newborns. But with unexpected high-risk admissions, hospitals need to call in highly specialized physicians regardless. Therefore, not letting the highly specialized physicians treat previously admitted lower-risk newborns and instead bringing in additional physicians with better-suited practice styles will incur additional costs. Hence, when taking into account the costs in optimizing physician–patient assignment, the spillover effects and hospitals' responses may be interpreted as a constrained optimization to accommodate fluctuations in care demand.

#### 3.2.3 Effect heterogeneity

Summary statistics in Table 1 indicates that care utilization and attending physician characteristics differ significantly across birth weight groups. If care providers consider birth weight as an important metric in making medical decisions, spillover effects of unscheduled high-risk admissions on incumbent newborns may also vary over birth weight. To flexibly trace out the distribution of spillover effects over birth weight, newborns with birth weight between 1,500 and 3,500 g are grouped into 100-g birth weight categories. Spillover effects at the high congestion level are estimated for each birth weight group.

Supplementary Figures 13–18 present the distribution of spillover effects over incumbent newborn birth weight. Although some estimates are less precise due to smaller sample sizes, all outcomes show consistent patterns: the effects become identifiable when birth weight drops below 2,300 g. In addition, newborns with birth weight near 1,500 g stay longer in the hospital and incur higher charges but do not experience different attending physicians practice styles, possibly because they are always treated by physicians specialized in high-risk cases. At the other end, zero effects on all outcomes are precisely estimated for newborns at the normal birth weight range, i.e., above 2,500 g.

The distribution of spillover effects over birth weight implies that not all incumbent newborns are equally likely to be assigned to the specialized physicians. To directly examine physician–patient assignment in the treated group, I compare incumbent newborns treated by the specialized physicians to incumbent newborns assigned to other physicians. If we hypothesize that newborns with relatively higher risks are assigned to the specialized physicians, incumbent newborns treated by other physicians would be positively selected in their health conditions. Empirical evidence well-supports this hypothesis (Supplementary Table 3). Treated group newborns assigned to the specialized physicians have a higher c-section rate, a lower fraction of females, and a significantly lower average birth weight. The difference in female fraction is driven by the gender difference in birth weight distribution where more male than female newborns are on the lower end of the birth weight distribution. On the other hand, treated group newborns assigned to other physicians are positively selected in their health condition with a lower c-section rate and higher birth weight. These results suggest that hospitals are aware of physician specialization and try to match physicians' skills to suited patient conditions to maximize productivity.

#### 3.2.4 Robustness checks

In this section, I show model robustness across different sets of covariates. I also test for alternative control groups and explore how the results change under different definitions of treated groups. Furthermore, I analyze healthy newborns, where we expect minimal spillover effects, as a placebo test. For all robustness analyses in this section, I only report results for the high congestion subsample where the spillover effects are prominent.

Results in Section 3.2 control for newborn observables including birth delivery method, insurance type, race and gender, and birth weight. If unscheduled high-risk admissions are indeed as good as random, then controlling for newborn observables should only increase estimation precision but not affect effect magnitudes. As a robustness check, stepwise regressions are implemented by adding control variables one at a time. Hospital-year, birth month, and birth day-of-week fixed effects are included in all regressions. Stepwise regression results show consistency across different sets of patient observable controls and carry similar magnitudes (Supplementary Table 4). R2 increases and standard error decreases when more control variables are included, generating more precise point estimates. Estimates on patient health outcomes stay small and insignificant (not reported).

Supplementary Figure 8 shows that newborns in the control group, i.e., admitted 3 or more days apart from unscheduled high-risk admissions, could still experience some hospital stay overlaps with unscheduled high-risk newborns. To reduce influence of unscheduled high-risk admissions on control group newborns and test result sensitivity, I adopt alternative control groups further away from unscheduled high-risk admissions and hold the treated group definition unchanged. Coefficient estimates are of comparable magnitudes when the control group consists of newborns admitted 4+ days, 5+ days, and 6+ days away from unscheduled high-risk admissions (Supplementary Table 5), showing that the potential spillover on control group newborns is negligible.

In addition, it is likely that not all newborns in the treated group are affected the same. To explore how effect magnitudes vary with different level of overlap with unscheduled high-risk newborns, alternative treated groups are defined to include newborn admissions: (1) within the 2 days prior (baseline), (2) 1 day prior, (3) 0–12 h prior, (4) 12–24 h prior, (5) 24–36 h prior, and (6) 36–48 h prior to unscheduled high-risk admissions (Supplementary Table 6).^10^ The group of mid-risk newborns admitted one prior to unscheduled high-risk admissions shows similar effect patterns as described in Sections 3.2.1 and 3.2.2. When examining spillover effects by incumbent newborn admission time in non-overlapping 12-h intervals, increase in care utilization is concentrated among newborns admitted 12–24 h prior to unscheduled high-risk admissions. Effects on attending physicians are mainly driven by newborns admitted 12–36 h prior, but are less precisely estimated.

Another possible source of variation in spillover effects is whether incumbent newborns stay inside or outside NICU. I compare effects among treated group newborns who are directly admitted to NICU after birth to those who are never admitted to NICU (Supplementary Table 7). Comparing effects among NICU and non-NICU incumbent newborns, all spillover effects on care utilization are concentrated among incumbent newborns inside NICU. No difference is seen in patient outcomes in either subgroup (not reported). It is worth noting that newborns with NICU admission are in worse heath conditions compared to non-NICU newborns. Hence, it is hard to pinpoint whether the heterogeneity is caused by newborn health conditions or NICU admission alone.

To complete the analysis, I investigate whether healthy incumbent newborns with birth weight of 2,500+ g are affected as a placebo test. Healthy newborns have limited demand for care after birth and stay mostly in regular nurseries that are physically separate from NICU facilities. Hence, unscheduled high-risk newborn admissions are expected to have little influence on low-risk newborns. With the advantage of larger sample size, all regression coefficients for the low-risk sample are precise zeros (Supplementary Table 8). This is consistent with the hypothesis that low-risk incumbent newborns have limited interaction with high-risk newborns and are hardly affected.

## 4 Discussion

The high and increasing health care spending in the United States and its unsatisfactory average health outcomes have long attracted the attention and from policy makers and researchers. At the same time, physicians are increasingly specialized in the United States, and their practice styles have been contributing to the high care utilization. Many questions centered at health care efficiency arise, such as whether more specialized physicians provide more effective health care service and what factors contribute to low return to care utilization. This paper examines an important however insufficiently studied topic: the role of physician–patient matching in measuring physician productivity and the returns to care, and it is the first to provide empirical evidence on hospitals' physician–patient matching decisions in response to demand uncertainty. When demand is unpredictable, i.e., hospitals cannot fully control the arrivals of patients, matching physician experts' skills to patient conditions they are best suited for requires frequent and recurring decision-making—an often-overlooked challenge in health care production. This paper capitalizes on the availability of rich information on physicians and patients, treatment decisions, health outcomes, and the assignment of patients to physicians in the hospital discharge data to study the matching decisions in response to demand shocks arising from unscheduled high-risk admissions.

Empirical findings in this paper show that hospitals summon highly specialized physicians and reoptimize physician–patient assignment upon temporary increases in care demand. This leads to spillover effects on patients admitted prior to unscheduled high-risk admissions: when hospitals are congested, these incumbent patients are more likely to be attended by physicians with more intensive practice styles who specialize in treating high-risk cases, leading to increases in care utilization for these patients without any detectable improvement in outcomes. The low productivity of specialized physicians when performing less familiar tasks has important implications. Whereas, it seems almost certain that less specialized individuals would not perform as well as highly specialized experts at complex tasks, more specialized or highly trained experts are not better at all tasks. Instead, experts' productivity strongly depends on their task assignments, making good skill–task matching essential in highly specialized production. These findings point to one important cause of the low return to health care expenditure: there exists costs beyond the payments to physicians that arise from the mismatch between physician experts who have more intensive treatment style and patients who are most suitable for the intensive treatment style, and such costs are more substantial when hospitals are congested. It is reasonable to think that the costs in optimizing physician–patient assignment is higher when hospitals are more congested. Therefore, hospitals' “mismatch” decisions *could* be a constrained optimization to accommodate fluctuations in care demand: when a hospital need to bring in highly specialized physicians upon unexpected high-risk admissions, if the hospital is already congested where physicians with better suited practice styles for lower risk patients likely are fully occupied, it becomes a highly complex decision regarding whether to balance physicians' workload by assigning lower risk patients to the newly called-in highly specialized physicians or to increase the workload of physicians who have better suited practice styles but may have reached their maximum capacity. Either decision could incur costs in terms of (under the former decision) increased care utilization or (under the latter decision) deteriorated patient outcomes.

This paper contributes to the question of health care efficiency by empirically document that physician–patient mismatches can and do lead to “flat of the curve” care provision in the US health care system. However, the research setting and data available in this paper does not allow one to study the normative question: what would be hospitals' optimal decision. Future studies establishing a general equilibrium model addressing the question of (constrained) optimal decision on physician–patient assignment or using data on physician and hospital resource costs to empirically assess hospitals benefit-cost tradeoffs will provide valuable insights and policy implications into how hospitals can improve care utilization efficiency by adjusting physician–patient assignments under demand uncertainty. Moreover, studies examining related questions using data from other countries will shed lights on how widely spread this challenge is across different health care systems and what remedies may be applicable to the US health care system if some countries are found more efficient in dealing such challenge.

## Data availability statement

This study utilizes limited-access data from New York State Department of Health Statewide Planning and Research Cooperative System (SPARCS). Data access requests are reviewed and approved by SPARCS Operations staff. Requests to access these datasets should be directed to https://www.health.ny.gov/statistics/sparcs/access/.

## Author contributions

The author confirms being the sole contributor of this work and has approved it for publication.

## Footnotes

^1^C-section births likely are scheduled. Despite some C-sections maybe unscheduled due to emergent medical conditions, those likely to only account for a small fraction of C-sections and cannot be separately identified in the data.

^2^The daily NICU patient census is derived based on the universe of patient admissions, regardless of whether they are in my newborn birth sample. Since the hospital discharge data in 2005 include patients who were admitted in 2004 and discharged in 2005, the NICU daily occupancy measure is precise from the 1st day of 2005.

^3^This pattern is recognized by the New York State Department of Health: “It has come to the New York State Department of Health (Department)'s attention that bed capacity in New York State neonatal intensive care units (NICU) is being exceeded on a frequent basis.” See DOH 2016 announcement, available at: https://www.health.ny.gov/professionals/hospital_administrator/letters/2016/2016-09-27_dal_16-14_nicu_vercrowding.htm.

^4^Newborns admitted on the same day or on the 2 days following an unscheduled high-risk admission are excluded from the main analyses. These newborns may experience very different spillover effects compared to incumbent newborns. Their admissions can also be endogenous to the unscheduled high-risk admissions.

^5^The same-day discharges are either transferred to a different medical facility or too sick to receive any medical care.

^6^A patient's attending physician can be different from the admitting physician. The attending physician is the one making most treatment decisions and can be assigned later during the stay. It is not unusual that a physician gets in hospital and receives a patient admitted on the previous day.

^7^Supplementary Table 1 reports estimated differences in attending physician practice style residual measures. Point estimates show a similar pattern when compared to Table 4.

^8^See estimated effect on cumulative care utilization in Supplementary Table 2.

^9^In-hospital mortality does not capture infant deaths outside hospitals. The 1-year in-hospital mortality is computed by tracing all hospital admissions of a newborn during the first year after birth. The in-hospital mortality is 0.7%, and the cumulative 1-year in-hospital mortality is 0.78% in my mid-risk sample. To benchmark the mortality measures in this paper, the overall 28-day and 1-year infant mortality in 2005–2009 U.S. Linked Birth/Infant Death Cohort Data is 0.45 and 1.4% among newborns with birth weight between 1,500 and 2,500 g.

^10^More than 70% of newborns in the treated group (3) and around 20% of newborns in the treated group (4) are admitted on the same day of unscheduled high-risk admissions, hence are not included in the baseline analyses. Newborns admitted within 12 h prior to unscheduled high-risk admissions are likely to have endogeneity issues because hospitals might be aware of the incoming high-risk newborns and pre-respond to the demand shocks.
