# Supplementary material for: The unexpected costs of expertise: evidence from highly specialized physicians
Source: Front Public Health. 2024 Mar 4;12:1108254. doi: 10.3389/fpubh.2024.1108254 (PMC10946670; doi:10.3389/fpubh.2024.1108254)
Supplement: Supplementary file 1 [file Presentation_1.pdf]

Supplemental Materials

S1 Data Description and Sample Construction

Statewide Planning and Research Cooperative System (SPARCS) is a comprehensive all payer data reporting system that collects information on discharges from hospitals. All facilities certified to provide inpatient services, ambulatory surgery services, emergency department services or outpatient services are required to submit data to SPARCS, including patient level detail on patient characteristics, diagnoses and treatments, services, and charges for each hospital inpatient stay. SPARCS data report patient age in days for patients younger than one year old. The principal diagnosis codes for newborns provide information on multiple births and birth delivery methods. I include all observations with a newborn principal diagnosis code and an age zero day in my sample of birth admissions. Birth weight is reported in grams for all newborn admissions. I assign a low birth weight (LBW) indicator to newborns with birth weight between 1500 and 2500 grams and a very low birth weight (VLBW) indicator to newborns with birth weight below 1500 grams.

The unique patient identifier (UPI) allows me to define two binary readmission variables : neonatal readmission is defined as having readmissions within 28 days after birth, and one-year readmission is defined as having readmissions within one year after birth. Length of hospital stay, total charges, and number of procedures received are aggregated across admissions during the first year after birth, including one's birth admission, to measure one-year cumulative care utilization. These outcome measures are matched to each newborn's birth admission record. Only the birth admission record for each newborn is included in the analysis sample.

The date of licensure listed on physician license is used to calculate physician tenure. A very small fraction of attending doctors in the newborn sample are not licensed physicians or were licensed outside of New York State, for whom no license profile can be matched. The missing rate is below 3%. I allow attending physician characteristics to evolve over time. For each patient admission, physician tenure is computed as the number of years between the year of admission and the year of physician licensure.

Since physician specialty is not listed on the license, I define a measure of "experience with at-risk newborns" as a proxy for physician expertise. For patients admitted on day t attended by physician p, the physician expertise is measured by the fraction of newborn patients being VLBW and LBW among all newborn patients attended by physician p up to day $t-1$.

In addition, I develop physician practice style baseline measures using average total charges, length of stay, and number of procedures among newborn patients *discharged* up to day $t-1$ by physician p. The averages are only measured up to the day before one's admission, hence eliminating any influence from the patient's own admission or future admissions. These baseline measures allow physicians' practice styles to evolve with the patient conditions they treated in the past.[1] To account for patient–physician selection, residual total charges, length of stay, and number of procedures are generated controlling for hospital-year fixed effects and patient observables. These residual measures capture physician practice style conditional on patient observables and hospital-year specific effects, hence may be interpreted as physician "intrinsic" styles. However, due to the presence of patient–physician selection, the effects of patient observables cannot be well identified. This will result in biased physician residual measures.[2] Therefore, I take raw averages as

[1] A physician with high average in total charges could be due to spending more on an average patient or treating more high-spending patients.

[2] To illustrate, if physicians with intensive practice styles always treat high-risk patients, then the effect of practice style cannot be separated from the effect of patient condition.

the preferred physician measures in this study.

S2 Possible Mechanisms of Spillover Effects

Results in Section 3.2.2 show that newborns admitted prior to unscheduled high-risk admissions are treated by physicians with more intensive practice styles and receive increased medical care. One plausible explanation is that the increase in treatment intensity is driven by physicians' practice styles unrelated to patient conditions. Hence, little benefit is observed in newborn health.

To explore the impact of physicians' practice styles, I follow Baron and Kenny (1986) to test a mediation hypothesis. Specifically, I seek to decompose the total spillover effects into direct effects that are not associated with the attending physicians' practice styles and indirect effects that are mediated through physicians' practice styles. Partial mediation occurs if the mediator variable accounts for some, but not all, of the total spillover effects.

Denoting the measure of physicians' practice styles as variable Phy, two regression equations are estimated simultaneously following the variable notation in equation (2):

$$\begin{aligned} Phy_{i,h,t} &= \phi_1 \cdot Pre_{i,h,t} + \beta X_{i,h,t} + \mu_{i,h,t}, \\ Y_{i,h,t} &= \phi_2 \cdot Pre + \theta \cdot Phy_{i,h,t} + \beta X_{i,h,t} + \epsilon_{i,h,t}. \end{aligned}$$

ϕ_2 measures the direct effect of Pre on Y and $\phi_1 \cdot \theta$ measures the indirect effect through physicians' practice styles, i.e., the mediator variable Phy.[3] The total effect is the summation of direct and indirect effects $(\phi_1 \cdot \theta + \phi_2)$.[4]

Appendix Table S9 column 1,3, and 5 report coefficients estimated simultaneously from the above two equations using structural equation modeling (SEM). The physician practice baseline measures defined in S1 are tested as mediator variables Phy. The direct effect is measured by the regression coefficient ϕ_2. The indirect and total effects are calculated by "nonlinear combinations of estimators" function *nlcom* where the standard errors are computed using the Delta Method. Indirect % measures the indirect fraction of total effect, i.e., the fraction through mediator variable Phy. Columns 2, 4, and 6 report the total spillover effects estimated from equation (2), which differ slightly from Table 5 due to the exclusion of observations missing physician practice measures. The indirect effect percentages reported in the bottom panel imply that physicians' practice styles account for 25% - 40% of the total increases in care utilization. Considering that the mediating variable Phy only captures one dimension of physician practice, the decomposed indirect effects through physician style can be interpreted as a lower bound. There could also exist spillovers in treatment practice across physicians. These effects are closely related to physician practice, but will be captured as direct effects instead of indirect effects through physician practice style measure Phy.

To further explore how physician styles affect care utilization, I test whether procedures performed on the unscheduled high-risk newborns increase the probability of receiving the same procedures among incumbent newborns patients. Specifically, I analyze three ICD-9 procedures that high-risk newborns frequently receive on the day of admission and are also common among mid-risk newborns: 93.90 non-invasive mechanical ventilation, 99.15 parenteral infusion of concentrated nutritional substances, and 99.83 other

[3]For patient i admitted on date t attended by physician p, $Phy_{i,h,t}$ is calculated to be average Y of patients discharged by the physician p up to date $(t-1)$.

[4]Although the Baron and Kenny (1986) method is the most commonly used approach in testing mediation effects and some associated technical issues can be addressed by structural equation modeling (SEM), it may suffer identification issues and the effect decomposition needs to be interpreted with caution (MacKinnon et al., 2007; Hayes, 2009; Zhao et al., 2010).

phototherapy. A regression modified from equation (2) is implemented:

$$\begin{aligned} Y^j_{i,h,t} &= \gamma_0 \cdot Pre_{i,h,t} \times (Proc^j_{i,h,t} = 0) + \gamma_1 \cdot Pre_{i,h,t} \times (Proc^j_{i,h,t} = 1) \\ &+ \beta X_{i,h,t} + \tau_{h,y} + \tau_{dow} + \tau_{mon} + \epsilon_{i,h,t} \end{aligned} \tag{4}$$

- $Y^j_{i,h,t,s} = 1$ if patient i admitted on date t in hospital h receives procedure j during the 2 days after the admission day.
- $Pre_{i,h,t} = 1$ if patient i admitted on date t in hospital h encounters an unscheduled high-risk newborn within the 2 days the admission day.
- $Proc^j_{i,h,t} = 1$ the unscheduled high-risk newborn receives procedure j on the day of admission.
- γ_0 captures the spillover effect when newborns in the treated group encounter an unscheduled high-risk newborn who does not receiving procedure j on the day of admission.
- γ_1 captures the spillover effect when newborns in the treated group encounter an unscheduled high-risk newborn who receives procedure j on the day of admission.

Appendix Table S10 summarizes regression results on the three procedures that are common both among high-risk and mid-risk newborns. The odd columns summarize the regression results among all mid-risk newborns and the even columns report estimates at high congestion levels. Encountering an unscheduled high-risk newborn receiving one of the three procedures on the day of admission significantly increases the probability of receiving the same procedure among incumbent newborns. No similar increase in procedure use is observed when encountering an unscheduled high-risk newborn not receiving such procedure. Appendix Table S10 even columns present the estimated effects when NICUs are congested. Although the estimates are less precise due to the reduction in sample size, the point estimates are positive and take larger values compared to effect estimates when the unscheduled high-risk newborns receive no such procedures. This finding suggests a mechanism through physician practice in addition to changes in physician–patient matching: physicians' practice styles on incumbent newborns may be directly influenced by the presence of unexpected high-risk newborns. This dynamic pattern of physicians' practice styles has been shown in many medical studies that physicians may resort to the availability heuristic, i.e., previous or concurrent patient events, in making treatment decisions (Choudhry et al., 2006). One could further test for the extent of cross-physician spillovers by checking whether such mechanism mainly exists among incumbent newborns treated by the attending physician of unexpected high-risk newborns. However, such analysis is severely underpowered due to the insufficient sample size of the data.

Another possible explanation of minimal health improvement from the increased treatment intensity is that unscheduled high-risk admissions may have led to initial negative spillovers on previously admitted newborn patients. Hence, hospitals retain incumbent newborns for longer stays and/or treat them more intensively to compensate for the initial negative effects. To test the crowding-out hypothesis, I investigate whether unscheduled high-risk admissions lead to delays in standard procedures after birth such as vaccinations and hearing tests, the two most common procedures after birth. Appendix Table S11 report regression estimates from equation (2) in the entire mid-risk sample and by congestion levels. Columns 1 and 2 report linear probability model estimates. The top panel sample averages show that more than 60% and 40% of newborns receive hearing tests and vaccination before discharge. To investigate any possible delays, I adopt two measures in columns 3 - 6, the number of days before receiving the two procedures, and a binary variable concerning whether the procedure is performed within the first 4 days during the stay. If initial crowding-out occurs, we would expect a longer period before receiving the procedure or a lower probability of receiving the procedure within the first few days. The sample averages in columns 5 - 6 indicate that hearing tests and vaccination are performed typically on the 3rd and 4th day after birth. Since newborns in the treated group are admitted within the two days prior to unscheduled high-risk admissions, these two procedures are subject to any crowding-out effects if that exists. All regression coefficients in Appendix

Table S11 are small and insignificant, indicating that unscheduled high-risk admissions do not change the overall probability or result in initial delays of receiving these two common procedures among incumbent newborns. Hence, it is unlikely that initial crowding-out masks any health benefits from increased care utilization among newborns admitted prior to unscheduled high-risk admissions.

References

Baron, R. M. and Kenny, D. A. (1986), 'The moderator–mediator variable distinction in social psychological research: Conceptual, strategic, and statistical considerations.', *Journal of personality and social psychology* **51**(6), 1173.

Choudhry, N. K., Anderson, G. M., Laupacis, A., Ross-Degnan, D., Normand, S.-L. T. and Soumerai, S. B. (2006), 'Impact of adverse events on prescribing warfarin in patients with atrial fibrillation: matched pair analysis', *Bmj* **332**(7534), 141–145.

Hayes, A. F. (2009), 'Beyond baron and kenny: Statistical mediation analysis in the new millennium', *Communication monographs* **76**(4), 408–420.

MacKinnon, D. P., Fairchild, A. J. and Fritz, M. S. (2007), 'Mediation analysis', *Annu. Rev. Psychol.* **58**, 593–614.

Zhao, X., Lynch Jr, J. G. and Chen, Q. (2010), 'Reconsidering baron and kenny: Myths and truths about mediation analysis', *Journal of consumer research* **37**(2), 197–206.

S3 Appendix Tables

Table S1: Differences in Attending Physician Practice Style - Residual Measures

	(1)	(2)	(3)	(4)	(5)	(6)
Congestion	Avg. Res. Len. of Stay (log)		Avg. Res. Charges (log)		Avg. Res. # of Proc.	
All	0.00221		0.00555		0.0181*	
	(0.00355)		(0.00993)		(0.0101)	
Low	-0.00654	-0.00942	-0.0156	-0.0223	-0.00527	-0.0125
	(0.00611)	(0.00643)	(0.0166)	(0.0161)	(0.0239)	(0.0262)
Medium	0.00492	0.00550	0.0139	0.0142	0.0255	0.0267
	(0.00378)	(0.00388)	(0.0113)	(0.0105)	(0.0194)	(0.0200)
High	0.00809	0.00784	0.0159	0.0183	0.0323	0.0309
	(0.00630)	(0.00619)	(0.0170)	(0.0159)	(0.0204)	(0.0197)
Covariate x C	No	Yes	No	Yes	No	Yes
N	23535	23535	23535	23535	23535	23535
Y-mean	0.0622	0.0622	0.201	0.201	0.213	0.213

Standard errors in parentheses   Standard errors clustered at hospital level

[a] For patients admitted on day t attended by physician p, physician practice style is measured as residual averages among newborn patients discharged by physician p up to day t-1. Residuals are generated by controlling for birth hospital-year, birth month, birth day of week, birth delivery method, insurance type, race, gender, and birth weight. See S1.

[b] A small fraction of patients have missing physician practice measures because there is no previously discharged patients by their attending physicians.

Table S2: Differences in One-Year Care Utilization

Congestion	(1) 1-Year Total Len. of Stay (log)	(2)	(3) 1-Year Total Charges (log)	(4)	(5) 1-Year Total # of Proc.	(6)
All	0.00707		-0.00251		0.0304	
	(0.00999)		(0.0168)		(0.0534)	
Low	-0.0150	-0.0191	-0.00868	-0.0287	-0.0680	-0.0328
	(0.0201)	(0.0209)	(0.0287)	(0.0317)	(0.0601)	(0.0633)
Medium	-0.00465	-0.00160	-0.0422*	-0.0354	0.00314	-0.000685
	(0.0149)	(0.0145)	(0.0247)	(0.0234)	(0.0716)	(0.0659)
High	0.0653**	0.0672**	0.0975*	0.104*	0.236	0.227
	(0.0270)	(0.0298)	(0.0514)	(0.0536)	(0.144)	(0.177)
Covariate x C	No	Yes	No	Yes	No	Yes
N	23791	23791	23791	23791	23791	23791
Y-mean	1.915	1.915	9.502	9.502	2.837	2.837

Standard errors in parentheses Standard errors clustered at hospital level

Table S3: Differences in Newborn Observable Characteristics

	(1) C-section	(2) Birth Weight	(3) Female	(4) White	(5) Black	(6) Medicaid
Panel A: Overall Treated - Control Group Differences (same as in Table 2)						
Pre	-0.0152	-5.043	-0.00888	-0.00481	-0.00363	0.00387
	(0.0123)	(6.373)	(0.00822)	(0.00957)	(0.0115)	(0.00760)
Panel B: Treated - Control Group Differences by Whether Assigned to the Specialized Physicians						
$Pre \times (Spec = 1)$	0.0385**	-81.07***	-0.0569***	-0.0108	0.0250	0.0224
	(0.0165)	(16.05)	(0.0150)	(0.0119)	(0.0178)	(0.0269)
$Pre \times (Spec = 0)$	-0.0372**	26.05**	0.0108	-0.00236	-0.0153	-0.00372
	(0.0170)	(10.66)	(0.0117)	(0.0129)	(0.0152)	(0.0137)
N	23791	23791	23791	23791	23791	23791

Standard errors in parentheses Standard errors clustered at hospital level

[a] Hospital-year, month, and day of week fixed effects are included in measuring treated–control differences.
[b] Panel A reports overall differences between the treated and control group newborns. Panel B separately reports treated–control differences for incumbent newborns who are assigned to the specialized physicians and incumbent newborns who are assigned to other physicians.

Table S4: Alternative Specifications
(Congestion = High)

	(1)	(2)	(3)	(4)	(5)
Length of Stay (log)	0.0906**	0.102**	0.103**	0.110***	0.0731***
	(0.0405)	(0.0393)	(0.0391)	(0.0382)	(0.0264)
R^2	[0.041]	[0.102]	[0.111]	[0.123]	[0.449]
Total Charges (log)	0.129	0.147*	0.148*	0.163**	0.107**
	(0.0782)	(0.0726)	(0.0732)	(0.0717)	(0.0444)
R^2	[0.121]	[0.173]	[0.179]	[0.193]	[0.484]
# of Procedures	0.237*	0.256*	0.257*	0.286**	0.208
	(0.139)	(0.134)	(0.135)	(0.140)	(0.125)
R^2	[0.179]	[0.200]	[0.204]	[0.236]	[0.359]
Experience with VLBW	0.00631*	0.00668*	0.00655*	0.00709**	0.00606*
	(0.00339)	(0.00335)	(0.00337)	(0.00336)	(0.00314)
R^2	[0.171]	[0.177]	[0.181]	[0.184]	[0.248]
Physician Avg Len. of Stay	0.0437*	0.0467**	0.0456*	0.0497**	0.0409**
(log)	(0.0224)	(0.0221)	(0.0228)	(0.0220)	(0.0188)
R^2	[0.156]	[0.164]	[0.170]	[0.177]	[0.274]
Physician Avg Total Charges	0.0767*	0.0830*	0.0805*	0.0886**	0.0708**
(log)	(0.0430)	(0.0421)	(0.0430)	(0.0414)	(0.0346)
R^2	[0.226]	[0.234]	[0.242]	[0.248]	[0.342]
Physician Avg # of Procedure	0.0896**	0.0952**	0.0937**	0.0990**	0.0849**
	(0.0382)	(0.0380)	(0.0386)	(0.0391)	(0.0348)
R^2	[0.436]	[0.441]	[0.443]	[0.446]	[0.498]
Delivery Method	No	Yes	Yes	Yes	Yes
Insurance Type	No	No	Yes	Yes	Yes
Race & Gender	No	No	No	Yes	Yes
Birth Weight (250g bin)	No	No	No	No	Yes
N	4986	4986	4986	4986	4986

Standard errors in parentheses, R^2 in square brackets   Standard errors clustered at hospital level

[a] All regressions include hospital-year, birth month, and day of week fixed effects

7

Table S5: Alternative Control Groups
(Congestion = High)

	(1)	(2)	(3)	(4)
Length of Stay (log)	0.0731***	0.0738***	0.0788***	0.0784***
	(0.0264)	(0.0247)	(0.0249)	(0.0260)
Total Charges (log)	0.107**	0.111**	0.128**	0.132**
	(0.0444)	(0.0469)	(0.0520)	(0.0548)
# of Procedures	0.208	0.171	0.177	0.212*
	(0.125)	(0.116)	(0.120)	(0.122)
Experience with VLBW	0.00606*	0.00571	0.00696*	0.00728*
	(0.00314)	(0.00362)	(0.00366)	(0.00368)
Physician Avg Len. of Stay	0.0409**	0.0369	0.0469*	0.0483*
(log)	(0.0188)	(0.0226)	(0.0245)	(0.0255)
Physician Avg Total Charges	0.0708**	0.0661	0.0850*	0.0886*
(log)	(0.0346)	(0.0421)	(0.0451)	(0.0456)
Physician Avg # of Procedures	0.0849**	0.0772*	0.0930**	0.100**
	(0.0348)	(0.0394)	(0.0427)	(0.0449)
Control Group	3+ Days Apart	4+ Days Apart	5+ Days Apart	6+ Days Apart
N	4986	4598	4250	3978

Standard errors in parentheses Standard errors clustered at hospital level

[a] Alternative control groups are defined in Section 3.2.4.

Table S6: Alternative Treated Groups
(Congestion = High)

	(1)	(2)	(3)	(4)	(5)	(6)
Length of Stay (log)	0.0731***	0.0773***	0.0662	0.168***	-0.0266	0.0240
	(0.0264)	(0.0269)	(0.0755)	(0.0549)	(0.0615)	(0.0491)
Total Charges (log)	0.107**	0.110**	0.121	0.243***	0.00886	0.00991
	(0.0444)	(0.0488)	(0.123)	(0.0819)	(0.133)	(0.0675)
# of Procedures	0.208	0.227	0.424	0.657***	-0.145	0.0947
	(0.125)	(0.140)	(0.276)	(0.202)	(0.151)	(0.192)
Experience with VLBW	0.00606*	0.00575	-0.00119	0.00663*	0.0113	-0.00453
	(0.00314)	(0.00407)	(0.00536)	(0.00388)	(0.00899)	(0.00491)
Physician Avg Len. of Stay	0.0409**	0.0530	-0.0145	0.0384	0.0920	-0.0516
(log)	(0.0188)	(0.0337)	(0.0587)	(0.0398)	(0.0671)	(0.0339)
Physician Avg Total Charges	0.0708**	0.0956	-0.0182	0.0470	0.190	-0.0910
(log)	(0.0346)	(0.0611)	(0.118)	(0.0739)	(0.134)	(0.0736)
Physician Avg # of Procedures	0.0849**	0.103	-0.000584	0.0726	0.151	-0.114*
	(0.0348)	(0.0644)	(0.102)	(0.0694)	(0.107)	(0.0560)
Treated Group	1-2 Days	1 Day	0-12 Hrs	12-24 Hrs	24-36 Hrs	36-48 Hrs
N	4986	4768	4670	4661	4679	4657

Standard errors in parentheses Standard errors clustered at hospital level

[a] Alternative treated groups are defined in Section 3.2.4.

Table S7: Effects among Patients inside and outside NICU
(Congestion = High)

Treated Group	(1) Len. of Stay (log)	(2) Total Charges (log)	(3) # of Procedures	(4) Experience with VLBW	(5) Avg. Len. of Stay (log)	(6) Avg. Total Charges (log)	(7) Avg. # of Procedures
All	0.0695**	0.118**	0.200	0.00657*	0.0522**	0.0964**	0.0997**
	(0.0293)	(0.0490)	(0.127)	(0.00376)	(0.0216)	(0.0400)	(0.0386)
Inside NICU	0.104**	0.181***	0.302*	0.0100	0.0667	0.112	0.137
	(0.0471)	(0.0500)	(0.165)	(0.00842)	(0.0445)	(0.0873)	(0.0866)
Outside NICU	-0.0165	-0.0770*	-0.0288	-0.00127	-0.00345	-0.00470	-0.00532
	(0.0260)	(0.0408)	(0.0943)	(0.00520)	(0.0367)	(0.0739)	(0.0662)

Standard errors in parentheses Standard errors clustered at hospital level

[a] Inside NICU: Newborns who are directly admitted to NICU after birth.
[a] Outside NICU: Newborns who are never admitted to NICU during the hospital stay.

Table S8: Placebo Test - Low-risk Newborn Sample

Congestion	(1)<br>Len. of Stay (log)	(2)<br>Total Charges (log)	(3)<br># of Procedures	(4)<br>Physician Tenure	(5)<br>Experience with VLBW	(6)<br>Avg. Total Charges (log)
Low	0.00334	0.00613	0.0117	-0.0952	0.000561	0.0122
	(0.00227)	(0.00600)	(0.0110)	(0.131)	(0.000487)	(0.0120)
Medium	-0.00177	-0.00281	-0.000322	0.117	-0.000354	-0.00231
	(0.00229)	(0.00510)	(0.00794)	(0.213)	(0.000293)	(0.00999)
High	0.00165	-0.00342	-0.000951	0.212	-0.000720	-0.00959
	(0.00403)	(0.00875)	(0.0113)	(0.254)	(0.000572)	(0.0127)
N	398562	398562	398562	389269	396117	394191
Y-mean	1.257	8.269	1.524	16.77	0.0126	8.666

Standard errors in parentheses Standard errors clustered at hospital level

[a] Sample: Singleton newborns with birth weight of 2500 grams or above.

Table S9: Mediation Analysis - Physician Practice Style
(Congestion = High)

	(1)	(2)	(3)	(4)	(5)	(6)
	Length of Stay (log)		Total Charges (log)		# of Procedures	
$Phy = \alpha_1 + \phi_1 Pre + +\beta X + \mu$						
Pre	0.0409**		0.0708**		0.0849**	
	(0.0187)		(0.0345)		(0.0347)	
$Y = \alpha_2 + \phi_2 Pre + \theta Phy + \beta X + \epsilon$						
Pre	0.0590**	0.0780**	0.0686*	0.111**	0.157	0.217**
	(0.0261)	(0.0308)	(0.0408)	(0.0544)	(0.122)	(0.0995)
Phy	0.464***		0.593***		0.714***	
	(0.0311)		(0.0380)		(0.0478)	
Effect Decomposition						
Direct Effect	0.0590**		0.0686*		0.157	
(ϕ_2)	(0.0261)		(0.0408)		(0.122)	
Indirect Effect	0.0190**		0.0420**		0.0607**	
($\phi_1 \cdot \theta$)	(0.00882)		(0.0207)		(0.0260)	
Total Effect	0.0780***		0.111**		0.217*	
($\phi_2 + \phi_1 \cdot \theta$)	(0.0262)		(0.0432)		(0.126)	
Indirect %	0.243		0.380		0.279	
N	4942	4942	4942	4942	4942	4942

Standard errors in parentheses Standard errors clustered at hospital level

[a] For each outcome Y, the physician practice measure Phy is the physician baseline measure of average Y defined in S1, i.e., the average Y of patients discharged by the physician p up to date $(t-1)$.
[b] The standard errors of the indirect and total effects are calculated using the Delta method.

Table S10: Treatment Procedure Spillover

	(1)	(2)	(3)	(4)	(5)	(6)
Procedure	Ventilation		Nutrition		Phototherapy	
$Pre \times (Proc^j = 0)$	-0.00243	-0.00658	-0.00993	-0.00876	-0.0104	0.00196
	(0.00390)	(0.00807)	(0.00691)	(0.0145)	(0.00768)	(0.0160)
$Pre \times (Proc^j = 1)$	0.0253**	0.0103	0.0961***	0.103*	0.147***	0.115
	(0.0109)	(0.0244)	(0.0312)	(0.0529)	(0.0324)	(0.0775)
N	23791	4986	23791	4986	23791	4986
Y-mean	0.0217	0.0231	0.0741	0.0642	0.133	0.125
Congestion	All	High	All	High	All	High

Standard errors in parentheses Standard errors clustered at hospital level

[a] $Proc^j_{i,h,t} = 1$ if the unscheduled high-risk newborn receives procedure measured in the outcome variable on the day of admission.

 10.3389/fpuph.2024.1108254

Table S11: Delays in Common Procedures

Congestion	(1)<br>Hearing Tests	(2)<br>Vaccinations	(3)<br>Hearing Tests within 4 Days	(4)<br>Vaccinations within 4 Days	(5)<br># Days before Hearing Tests	(6)<br># Days before Vaccinations
All	0.00502	0.00691	0.00406	0.00202	0.0438	0.0722
	(0.00893)	(0.00603)	(0.00942)	(0.00777)	(0.0825)	(0.0904)
N	23791	23791	23791	23791	14574	10174
Y-mean	0.613	0.428	0.433	0.341	3.093	2.047
Low	-0.00550	0.00859	0.00833	0.00222	-0.118	0.0667
	(0.0106)	(0.0122)	(0.0138)	(0.0109)	(0.136)	(0.151)
N	7015	7015	7015	7015	4313	3042
Y-mean	0.616	0.435	0.429	0.345	3.162	2.051
Medium	0.00696	-0.00501	-0.00246	-0.00290	0.124	-0.0275
	(0.0124)	(0.00965)	(0.0127)	(0.0110)	(0.116)	(0.124)
N	11790	11790	11790	11790	7258	5063
Y-mean	0.616	0.430	0.435	0.342	3.094	2.071
High	0.00734	0.0224	-0.00187	0.00540	0.158	0.381
	(0.0190)	(0.0271)	(0.0197)	(0.0246)	(0.161)	(0.240)
N	4986	4986	4986	4986	2990	2044
Y-mean	0.601	0.413	0.435	0.333	2.993	1.979

Standard errors in parentheses Standard errors clustered at hospital level

[a] Hearing tests and vaccinations are the top two procedure categories performed on newborns after birth.

S4 Appendix Figures

Figure S1: Average Total Charges by Birth Weight

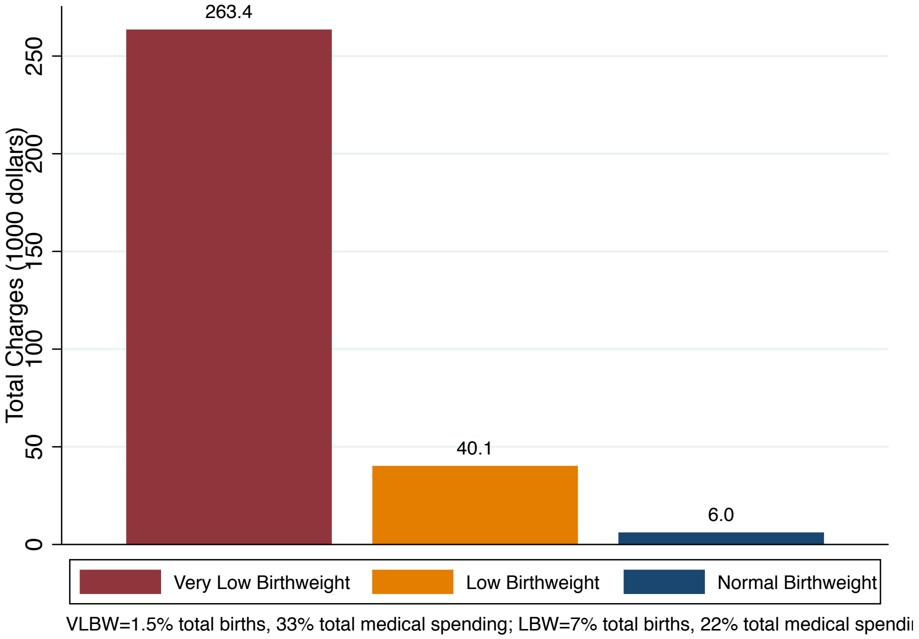

Figure S2: Probability of In-hospital Death by Birth Weight

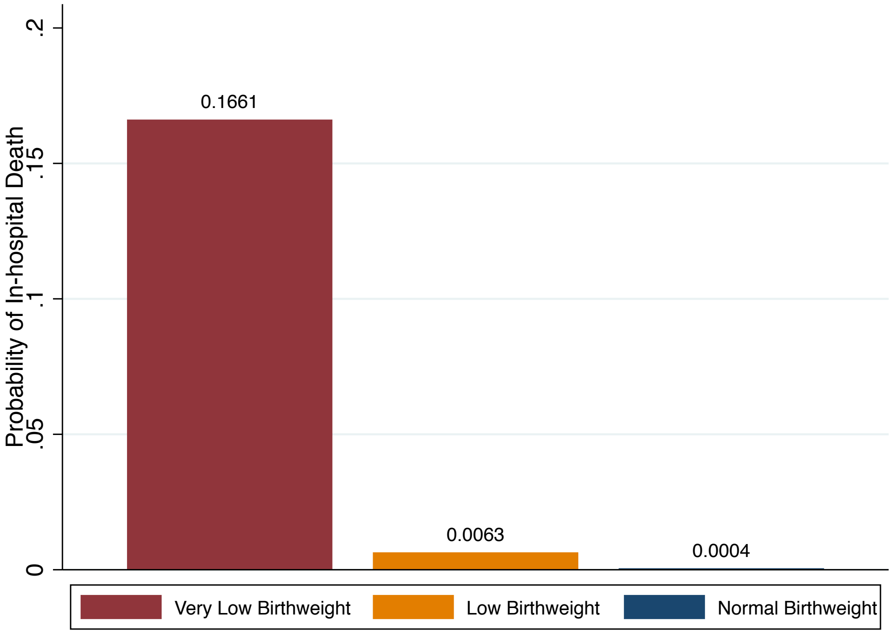

Figure S3: Distribution of Birth Weight

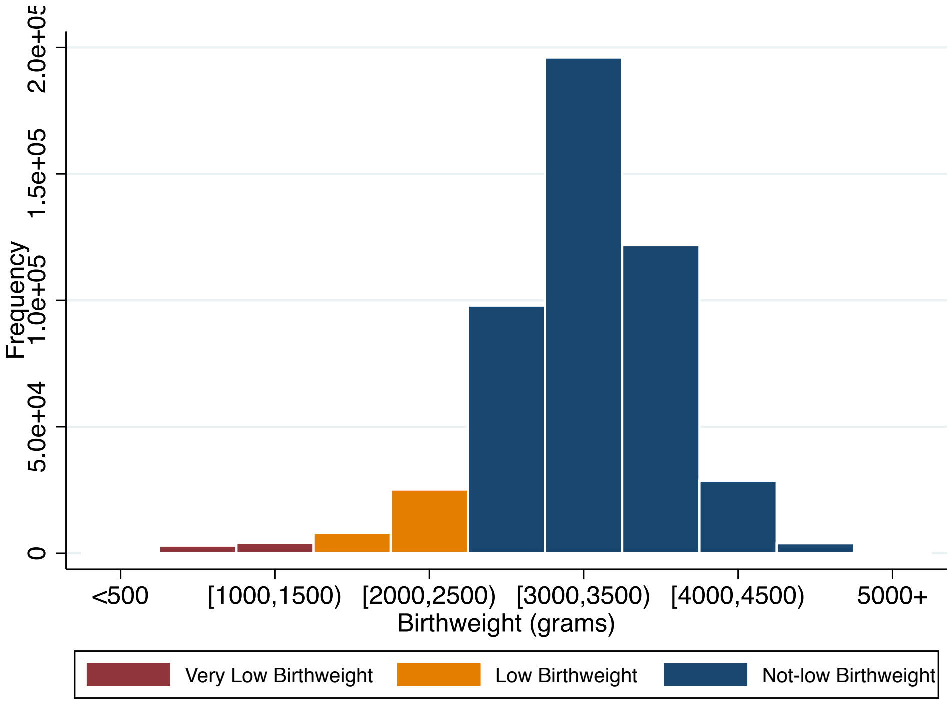

Figure S4: Physician Tenure by Birth Weight

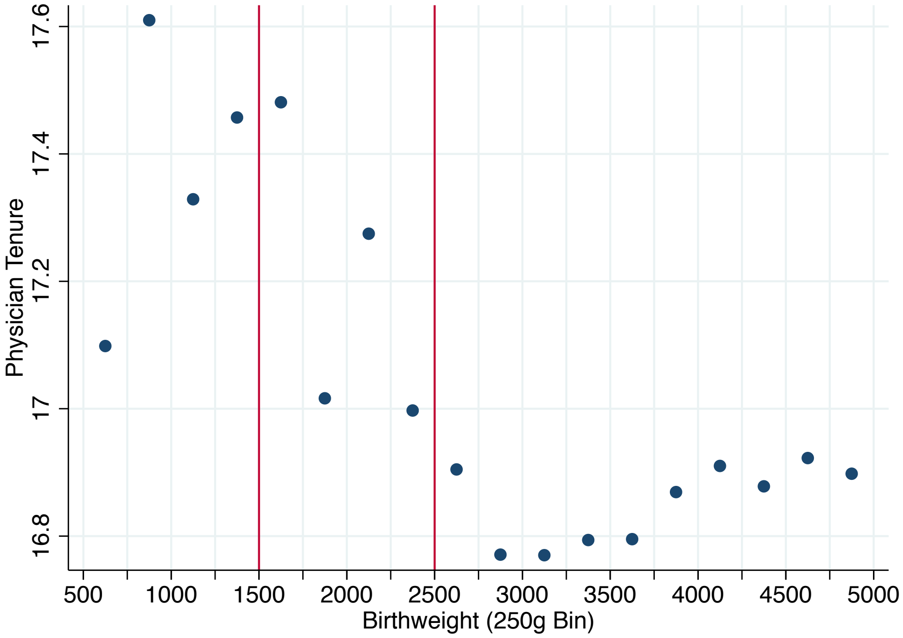

Newborns with birth weight below 500g or above 5000g are excluded.

Figure S5: Physician Experience with VLBW Patient by Birth Weight

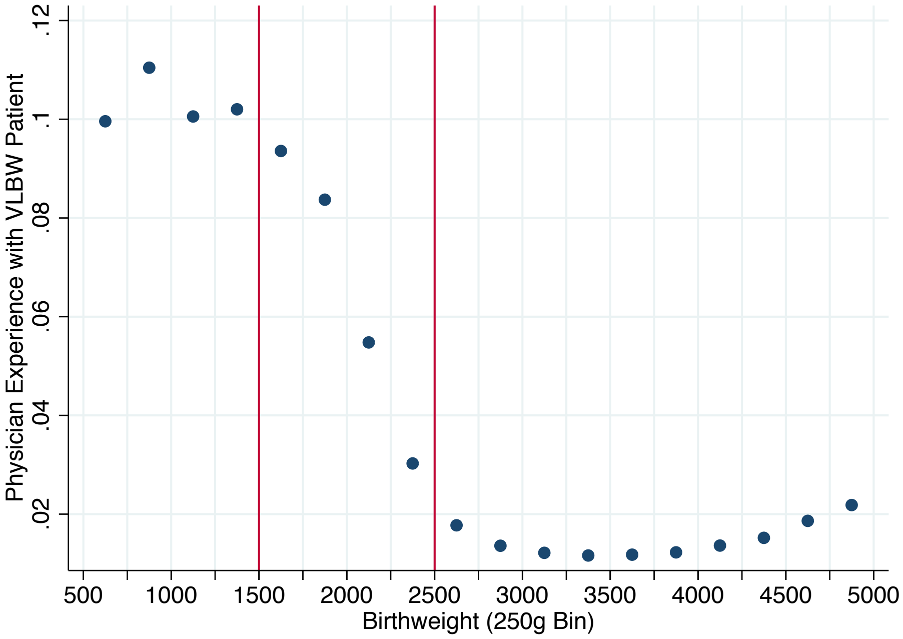

Newborns with birth weight below 500g or above 5000g are excluded.

Figure S6: Physician Style by Birth Weight - Baseline Measure

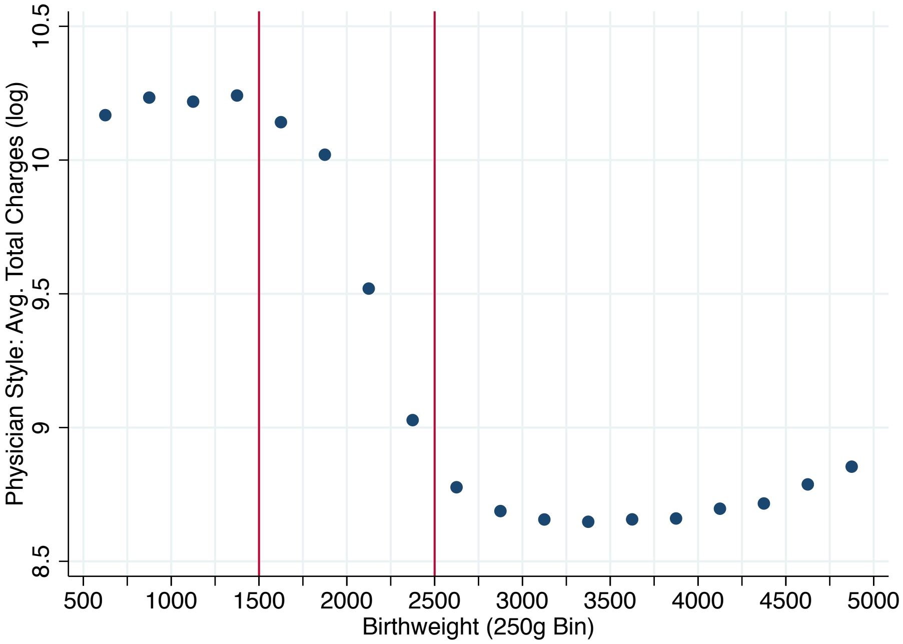

Newborns with birth weight below 500g or above 5000g are excluded.

Figure S7: Physician Style by Birth Weight - Residual Measure

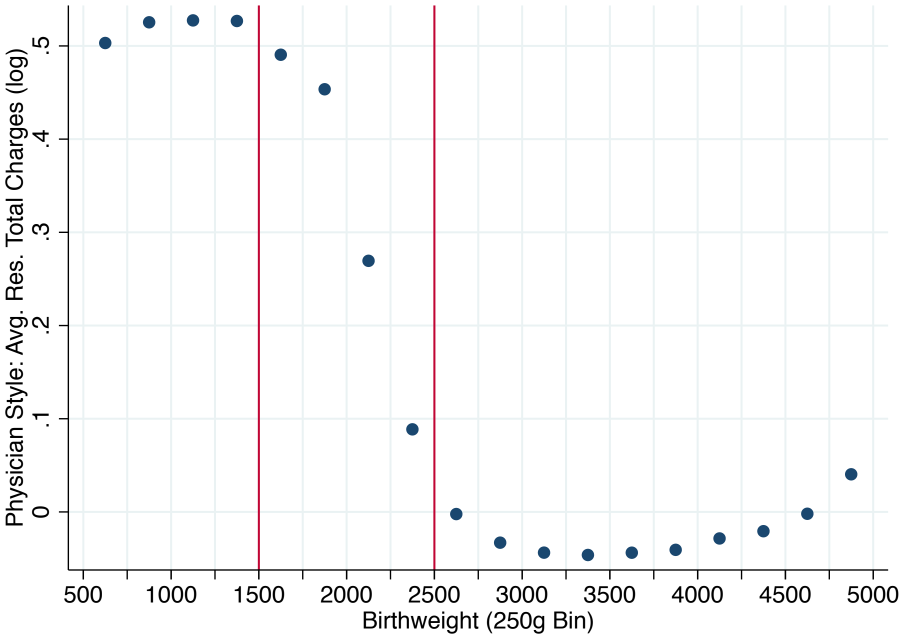

Newborns with birth weight below 500g or above 5000g are excluded.

Figure S8: Identifying a Treated Group by Overlap with High-risk Admission

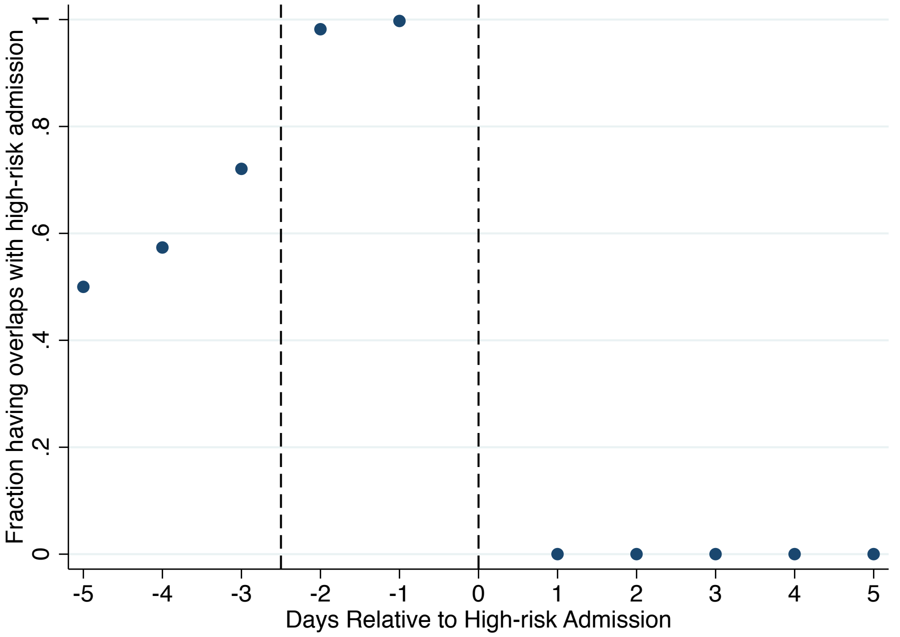

Figure S9: Distribution of Length of Hospital Stay

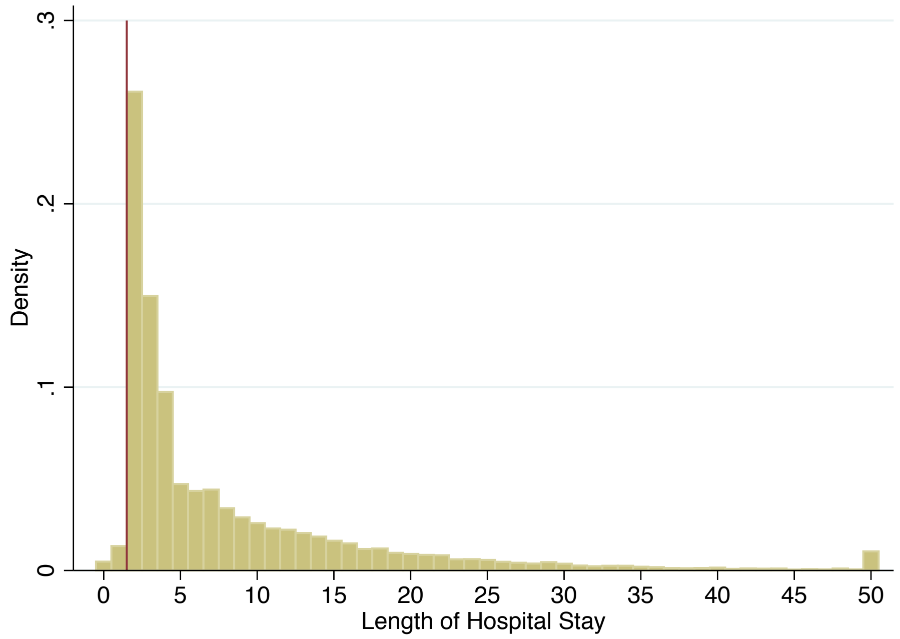

Sample: Singleton low birth weight newborns.

Figure S10: Distribution of Daily Procedures

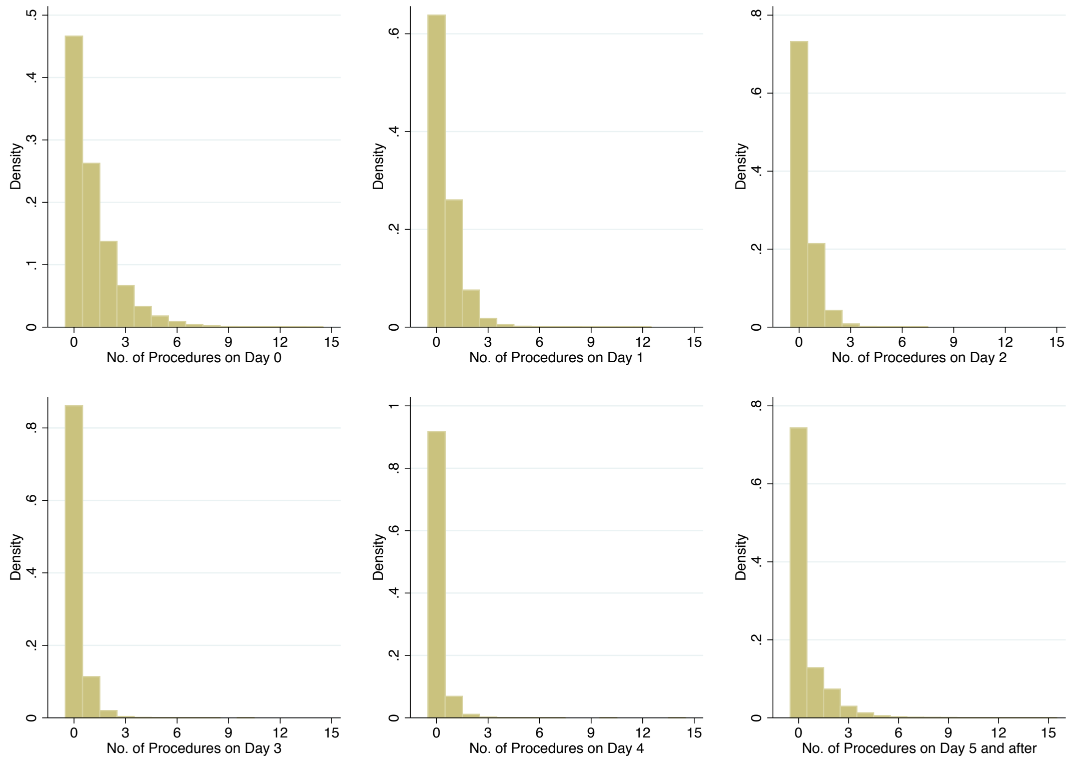

Sample: Singleton low birth weight newborns.

Figure S11: Daily Admissions and Discharges of NICU Patients

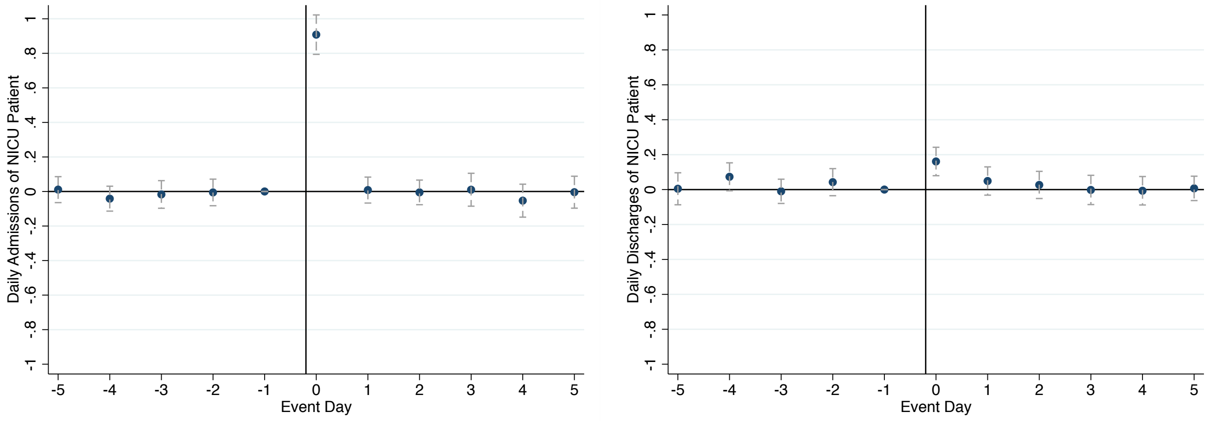

Figure S12: Daily Admissions and Discharges of NICU Patients

(high congestion on event day -1)

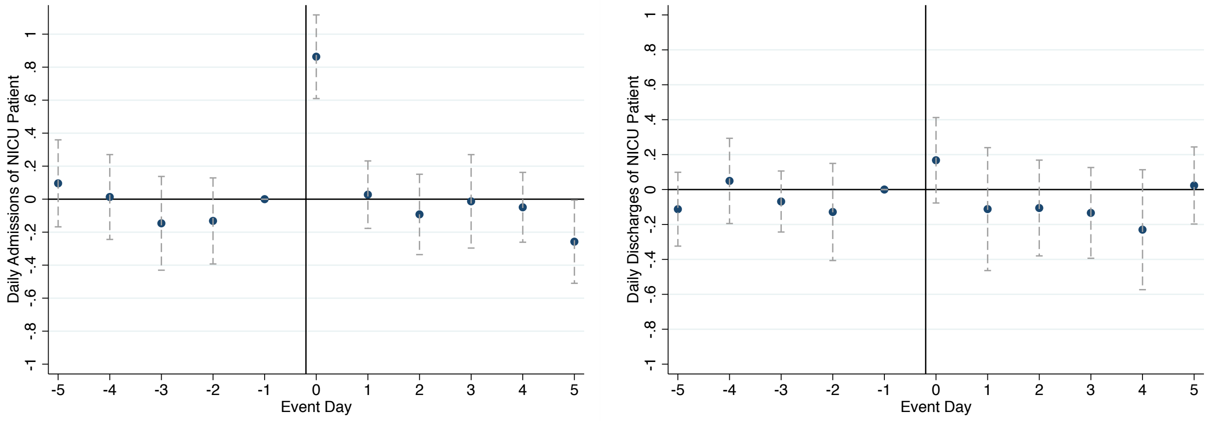

Graph generated by DD-event study to difference out the mean-reversion trend

Figure S13: Marginal Effect over Birth Weight

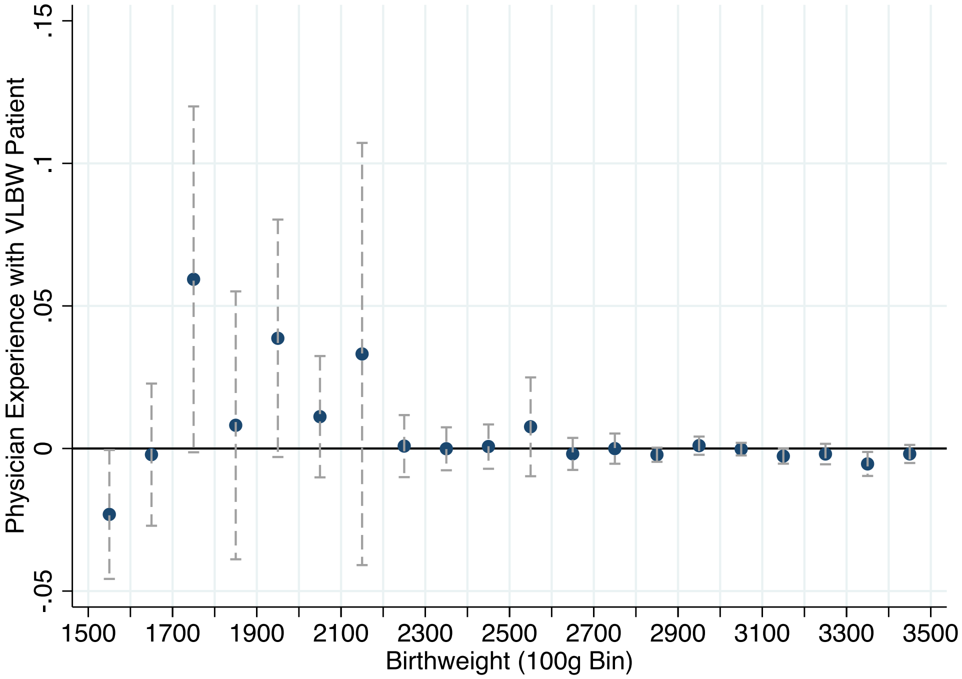

Figure S14: Marginal Effect over Birth Weight

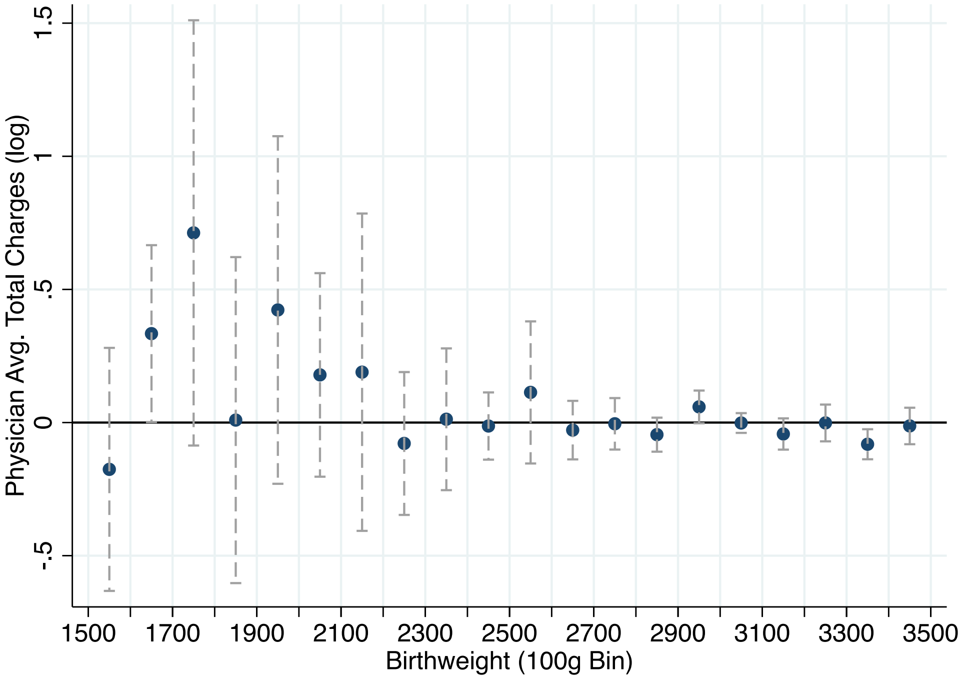

Physician average length of stay and number of procedures show similar distribution.

Figure S15: Marginal Effect over Birth Weight

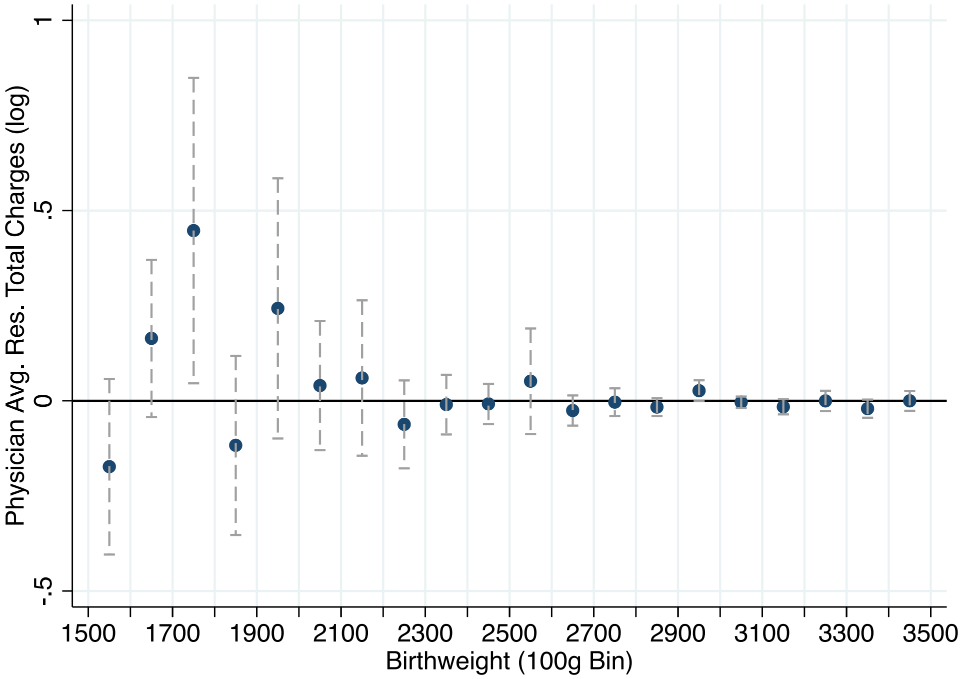

Physician average residual length of stay and number of procedures show similar distribution.

Figure S16: Marginal Effect over Birth Weight

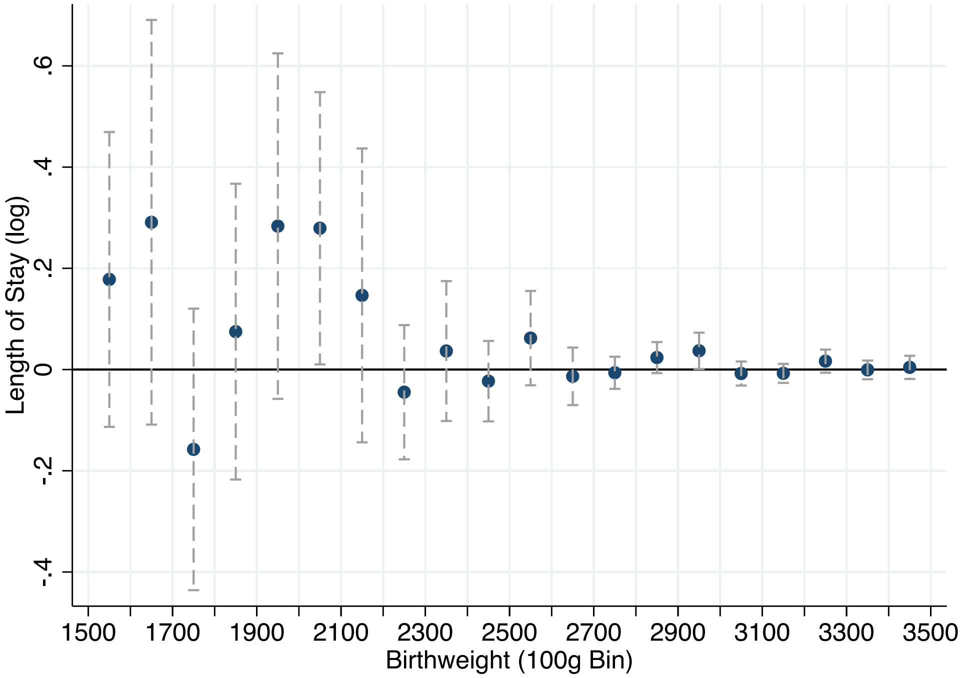

Figure S17: Marginal Effect over Birth Weight

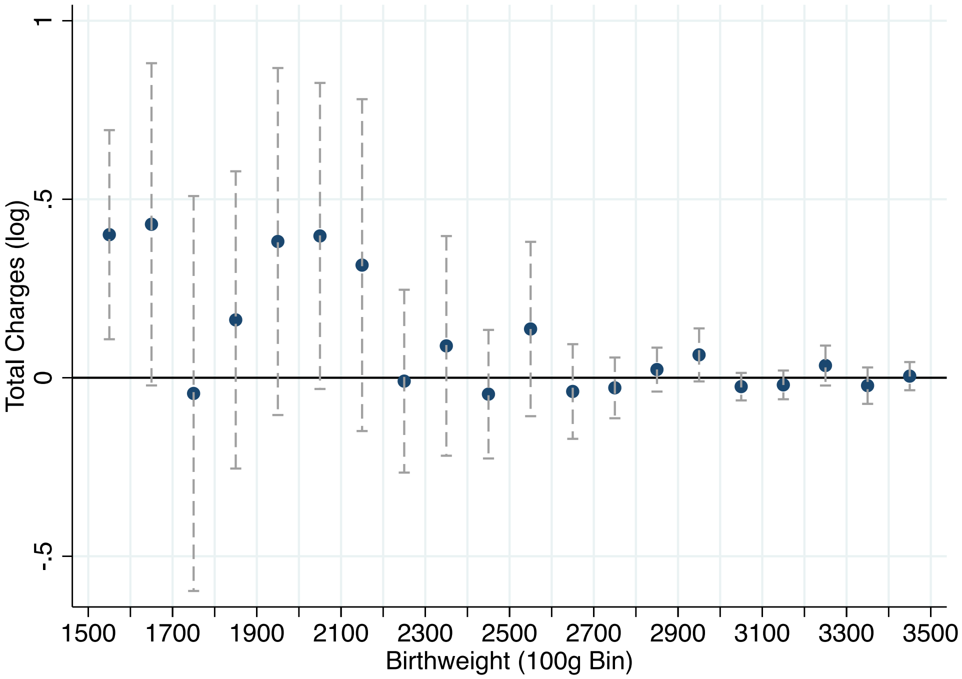

Figure S18: Marginal Effect over Birth Weight

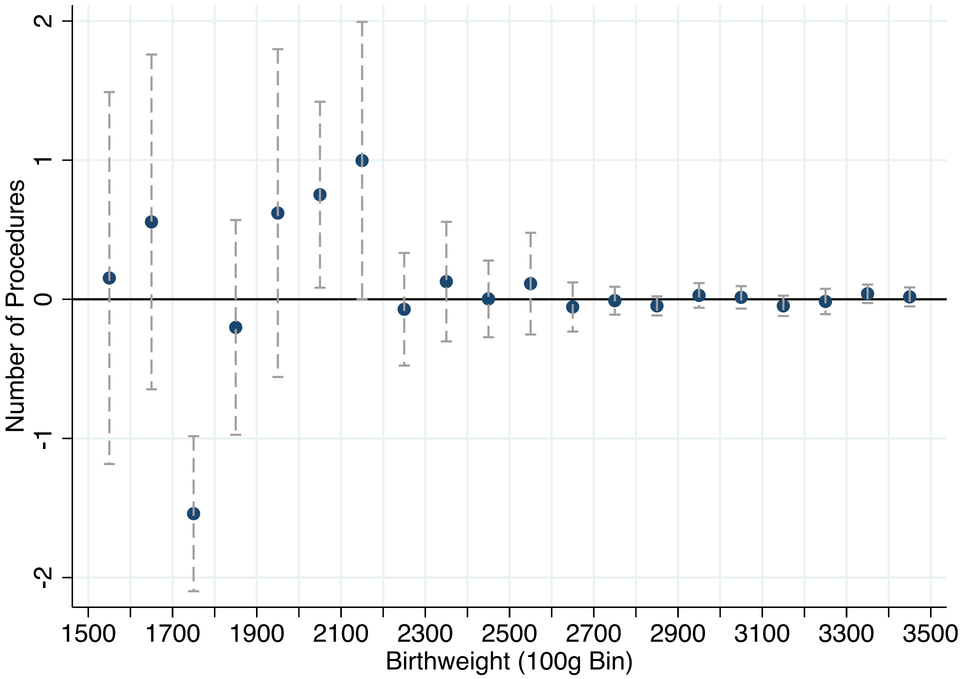

21
